# Orbit/CLASP Is Required for Myosin Accumulation at the Cleavage Furrow in *Drosophila* Male Meiosis

**DOI:** 10.1371/journal.pone.0093669

**Published:** 2014-05-21

**Authors:** Daishi Kitazawa, Tatsuru Matsuo, Kana Kaizuka, Chie Miyauchi, Daisuke Hayashi, Yoshihiro H. Inoue

**Affiliations:** Insect Biomedical Research Center, Kyoto Institute of Technology, Matsugasaki, Kyoto, Japan; Institut de Génétique et Développement de Rennes, France

## Abstract

Peripheral microtubules (MTs) near the cell cortex are essential for the positioning and continuous constriction of the contractile ring (CR) in cytokinesis. Time-lapse observations of *Drosophila* male meiosis showed that myosin II was first recruited along the cell cortex independent of MTs. Then, shortly after peripheral MTs made contact with the equatorial cortex, myosin II was concentrated there in a narrow band. After MT contact, anillin and F-actin abruptly appeared on the equatorial cortex, simultaneously with myosin accumulation. We found that the accumulation of myosin did not require centralspindlin, but was instead dependent on Orbit, a *Drosophila* ortholog of the MT plus-end tracking protein CLASP. This protein is required for stabilization of central spindle MTs, which are essential for cytokinesis. Orbit was also localized in a mid-zone of peripheral MTs, and was concentrated in a ring at the equatorial cortex during late anaphase. Fluorescence resonance energy transfer experiments indicated that Orbit is closely associated with F-actin in the CR. We also showed that the myosin heavy chain was in close proximity with Orbit in the cleavage furrow region. Centralspindlin was dispensable in Orbit ring formation. Instead, the Polo-KLP3A/Feo complex was required for the Orbit accumulation independently of the Orbit MT-binding domain. However, *orbit* mutations of consensus sites for the phosphorylation of Cdk1 or Polo did not influence the Orbit accumulation, suggesting an indirect regulatory role of these protein kinases in Orbit localization. Orbit was also necessary for the maintenance of the CR. Our data suggest that Orbit plays an essential role as a connector between MTs and the CR in *Drosophila* male meiosis.

## Introduction

Cytokinesis is facilitated by the constriction of the contractile ring (CR), which is composed of F-actin and myosin II fibers [1], [2]. The manner by which the CR is formed on the equatorial region of the cell cortex is not fully understood. Accumulating evidence has suggested that anaphase spindle microtubules (MTs) influence specification of the cleavage plane [3], [4], [5], [6]. It is generally believed that astral MTs play a negative role in the positioning of the cleavage furrow by sending an inhibitory signal that prevents CR formation at the cell equator [7], [8]. On the other hand, robust MT bundles, known as central spindle microtubules (CS MTs), are constructed between the separating chromosomes at late anaphase. These CS MTs provide a structural base from which a signal to initiate membrane ingression is sent to the prospective cleavage furrow (CF) region on the equatorial cortex [9]. Interdependency between the CS and the CR has been reported in cytokinesis [10], [11]. However, the molecular mechanism that mediates the linkage between the CS and the CR at late anaphase has not been fully elucidated.

One of the most important regulators that specify the cleavage plane is a protein complex known as centralspindlin, consisting of RacGAP50C and the *Drosophila* kinesin-6 protein, Pav. Centralspindlin is critical for both CS assembly and cytokinesis. It is associated with Pebble and targets this RhoGEF to the equatorial cortex [12]. Pebble mediates the conversion of GDP-Rho1 to GTP-Rho1 and activates Rho1-dependent cascades at the equatorial cortex [12], [13], [14], [15], [16]. Targeting of ECT2/Pebble to the cortex depends on the phosphorylation of MgcRacGAP/RacGAP50 by Plk1/Polo [17], [18], [19], [20]. The hypomorphic *polo* mutant of *Drosophila* showed defects in cytokinesis during male meiosis [21]. Pav and Polo interact and depend on each other for localization on the CS in *Drosophila* embryos [22]. The centralspindlin complex, consisting of RacGAP50C and Pav/MKLP-1, can localize the Pebble/RhoGEF on the equatorial cortex to initiate F-actin polymerization in order to construct the contractile ring [23].

In addition, anillin plays an important role in the initiation and progression of cytokinesis [24], [25], [26], [27], [28]. Anillin is known as a CR component that was first observed in the equatorial region in cultured *Drosophila* and mammalian cells [23], [26], [27]. Anillin binds F-actin, myosin II, and septins. This scaffold protein also binds Rho and RacGAP50C in somatic cells. Therefore, anillin has been considered as a key factor for maintenance of the actomyosin ring, which causes the ring to couple to CS MTs at anaphase [27], [29]. Furthermore, recent studies have reported that anillin depletion did not affect the recruitment of F-actin or myosin to the CF, although it was necessary for septin recruitment in yeast to mammalian cells [30], [31], [26], [32].

Several microtubule-associated proteins (MAPs) have also been described as essential factors for cytokinesis in *Drosophila* male meiosis [33], [34], [35]. Feo, the *Drosophila* ortholog of PRC1, is specifically enriched at the CS mid-zone and is required for cytokinesis in spermatocytes [34], [35]. Orbit, a *Drosophila* ortholog of the conserved MAP family known as CLASP, is essential for proper organization of mitotic spindles in early embryos, larval neuroblasts, and cultured cells [5], [36], [37]. The mammalian CLASP is enriched on the plus-ends of MTs and contributes to the stabilization of the ends in the cell cortex during interphase [38]. CLASP mediates multiple MT-based cellular events in both interphase and mitosis [37], [39]. Immunostaining data demonstrated that Orbit remains predominantly within the spindle envelope and associates with kinetochores and MT plus-ends during metaphase [33]. Upon entering anaphase, Orbit distributes along interior central spindle MTs and begins to concentrate in the mid-part of the CS, where it remains as cleavage continues [40], [33]. Orbit plays an essential role in cytokinesis during male meiosis. Although evidence of its direct interaction with the CR has not yet been obtained, the mouse ortholog CLASP2 can bind actin filaments by using unique sequences in interphase cells [41]. Fluorescence resonance energy transfer (FRET) experiments indicated that *Drosophila* Orbit is also closely associated with F-actin enriched in the fusome [42]. Nevertheless, several aspects of the role of Orbit in cytokinesis remain contradictory and unclear. Immunostaining conducted in a previous study did not demonstrate cortical localization of Orbit in spermatocytes during late anaphase, although mutants did show a defect in cleavage furrow ingression [33]. Time-lapse observation of the mutant spermatocytes showed that regression of CF ingression also occurred in cytokinesis. Although abnormal spermatocytes devoid of actomyosin rings or having disintegrated anillin rings were observed by immunostaining, it was not clear whether this was due to disturbance in CR formation or because the assembled CR could not be maintained [33]. Recently, it was reported that Orbit is required for germline cyst formation by the regulation of the germline-specific cytoskeleton fusome and ring canal, which is a bridge structure interconnecting germ line cells [42].

In this study, we examined the recruitment and assembly of CR components at the prospective CF region using time-lapse observations of living *Drosophila* spermatocytes. We found that myosin accumulation at the presumptive furrow was performed in two successive steps. Myosin was initially recruited to the cell cortex around the mid-zone. Shortly after the peripheral CS MTs made contact with the equatorial cortex to initiate CR formation, it became concentrated there in a band. Meanwhile, anillin and F-actin abruptly accumulated in a narrow band. We found that the initial recruitment of myosin was independent of MTs, but that the restriction step required MT structures. Interestingly, centralspindlin was dispensable in these steps. Instead, the accumulation of myosin required Orbit. The protein was localized in an overlapping region of peripheral MTs, and concentrated in a band on the equatorial cortex at late anaphase. Although centralspindlin was dispensable for the accumulation of Orbit, the Polo and KLP3A-Feo complex, which is essential for Polo recruitment, was required for Orbit accumulation. However, as *orbit* mutations of consensus amino acid sequences for phosphorylation by Cdk1 or Polo did not influence its accumulation, it appears that the Polo-mediated regulatory system plays an indirect role in Orbit localization. This protein was also necessary for the maintenance of the CR through its close association with myosin and F-actin in the CR. Our present data suggest that Orbit plays an essential role as a connector between MTs and the CR during cytokinesis in *Drosophila* male meiosis.

## Results

### Two-step accumulation of myosin II on the equatorial cell cortex at late anaphase in *Drosophila* male meiosis

**Figure 1:**
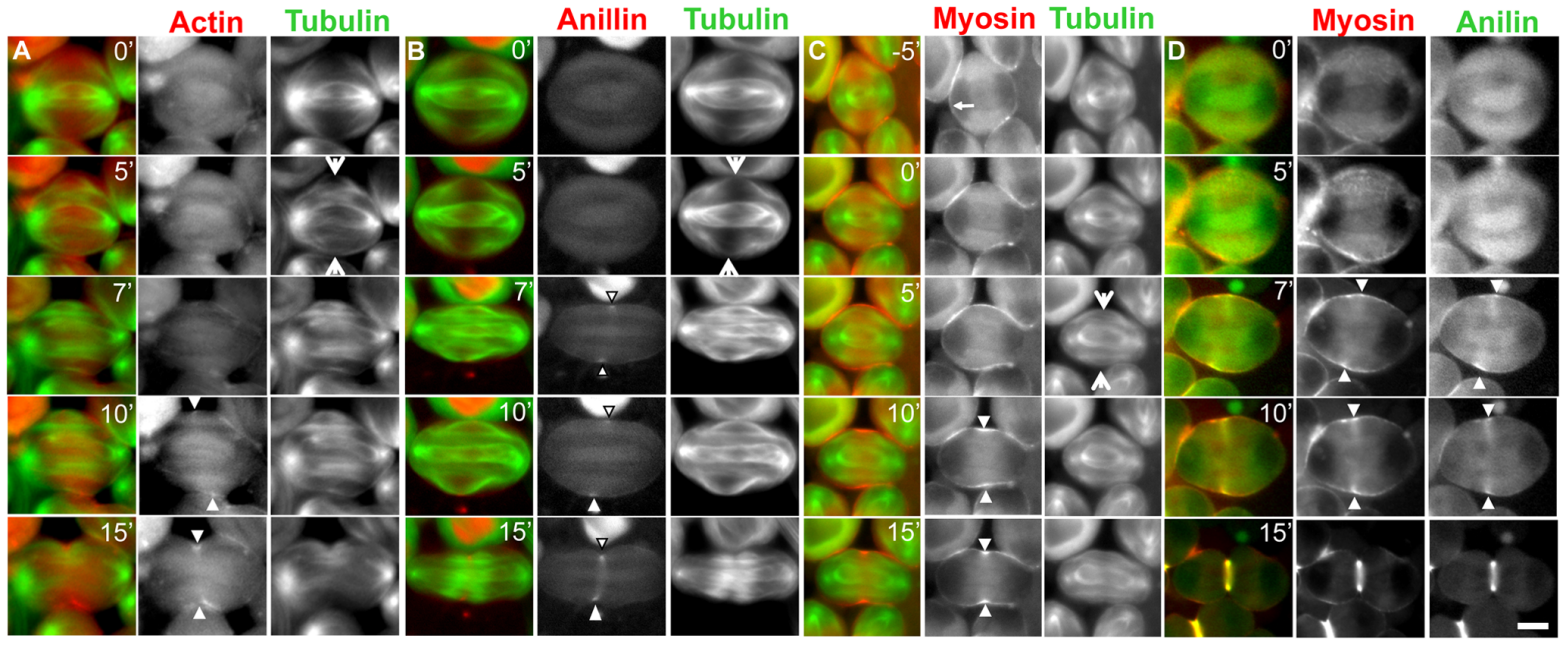
Accumulation of contractile ring components initiates at the cell cortex site where peripheral MTs contacted. (A) Time-lapse observation of microtubules and F-actin in primary spermatocytes with GFP-tubulin (green) and RFP-actin (red) expression during male meiosis. The peripheral CS MTs continued to probe the cytoplasm in the spermatocyte at anaphase onset (t = 0 min). The MTs emanating from opposite poles met together at the plus-end and made contact with the equatorial cortex (arrow, t = 5 min). Then, the anti-parallel MTs overlapped at the plus-end and initiated bundle formation. Five minutes after the MTs contacted the cortex, F-actin began to accumulate in a ring at the contact site (arrowheads, t = 10 min). (B)Time-lapse observation of RFP-anillin (red) and GFP-tubulin (green). After MTs established contact (arrow, t = 5 min), anillin began to accumulate in the site where the MTs contacted (arrowheads, t = 7 min). (C) Time-lapse observation of MTs and myosin in spermatocytes with RFP-tubulin (green) and GFP-Sqh (red) expression. Myosin distribution initially appeared along the cortex (small arrow, t = −5 min) before bundles of peripheral MTs formed. Then, soon after the peripheral MTs made contact with the cortex (large arrow, t = 5 min), a focus corresponding to the myosin ring appeared (arrowheads, t = 10 min). (D) Simultaneous observation of RFP-anillin (red) and GFP-Sqh (red). Abrupt recruitment of the anillin ring in the equatorial cell cortex (t = 7 min) was observed at the same time as the assembly of myosin that was recruited on the equatorial cortex (arrowheads). Scale bar  = 10 µm.

To understand the accumulation mechanism of CR components in the prospective CF region of the cell cortex, we simultaneously observed the dynamics of MTs and F-actin or myosin II during meiotic division in living *Drosophila* spermatocytes. Previous studies showed that the peripheral CS MTs make contact with the equatorial cortex and bundle together at mid-anaphase, and that shortly thereafter, CF ingression is initiated at the contact site [33]. We further performed simultaneous time-lapse observations of MTs with F-actin in living spermatocytes expressing green fluorescent protein (GFP)-tubulin and red fluorescent protein (RFP)-actin (Fig. 1A, Movie S1, Movie S2). The antiparallel peripheral MTs emanating from both spindle poles first met at the plus-ends and then made contact with the equatorial cortex (arrow, Fig. 1A). Five minutes after the peripheral MTs made contact, fluorescence foci of RFP-actin, corresponding to the F-actin ring, became visible (arrowheads, Fig. 1A). Similarly, anillin foci also appeared within 5 min after the MTs made contact (Fig. 1B).

On the other hand, distinctive foci of myosin II labeled with GFP-Sqh/myosin light-chain (MLC) could also be observed within 5 min of the MTs making contact with the cortex (Fig. 1C, Movie S3). Before the contact, the myosin was observed throughout the region of the cell cortex (small arrow, t = −5 min, Fig. 1C). This indicated that myosin had been recruited along the cortex before the MTs made contact with the equatorial cortex. Then, the already recruited myosin was accumulated within 5 min after contact with MTs (arrowheads, t = 10 min, Fig. 1C), as shown by the F-actin assembly results. Therefore, accumulation of myosin on the equatorial cortex can be separated into two successive steps of recruitment and accumulation. We confirmed that MLC and anillin are assembled at a similar time frame in the CF region (arrowheads, Fig. 1D, t = 10 min) and are co-localized on the contractile ring during cytokinesis. As the regulatory mechanism of myosin II dynamics during male meiosis is yet to be fully understood, we focused on myosin accumulation on the equatorial cortex in this study.

### Myosin II accumulation at the equatorial cortex is dependent on MTs but not on the centralspindlin complex

To elucidate the molecular mechanism of myosin II accumulation at the presumptive furrow in primary spermatocytes, we first investigated whether this accumulation was dependent on MTs. We examined the dynamics of Sqh/MLC in male meiosis in the presence of colchicine (Fig. S1B in File S1). We selected primary spermatocytes, colchicine was added at the onset of anaphase, and the dynamics of MTs and myosin were continuously observed. Myosin distribution temporarily appeared along the cell cortex (t = 5 min, arrows in Fig. S1B in File S1). The myosin failed to accumulate into single foci around the equatorial cortex (open arrowheads, Fig. S1B in File S1). This demonstrated that MTs are critical for the accumulation of myosin II in the prospective CF region, although they are dispensable for its initial recruitment along the cell cortex.

**Figure 2:**
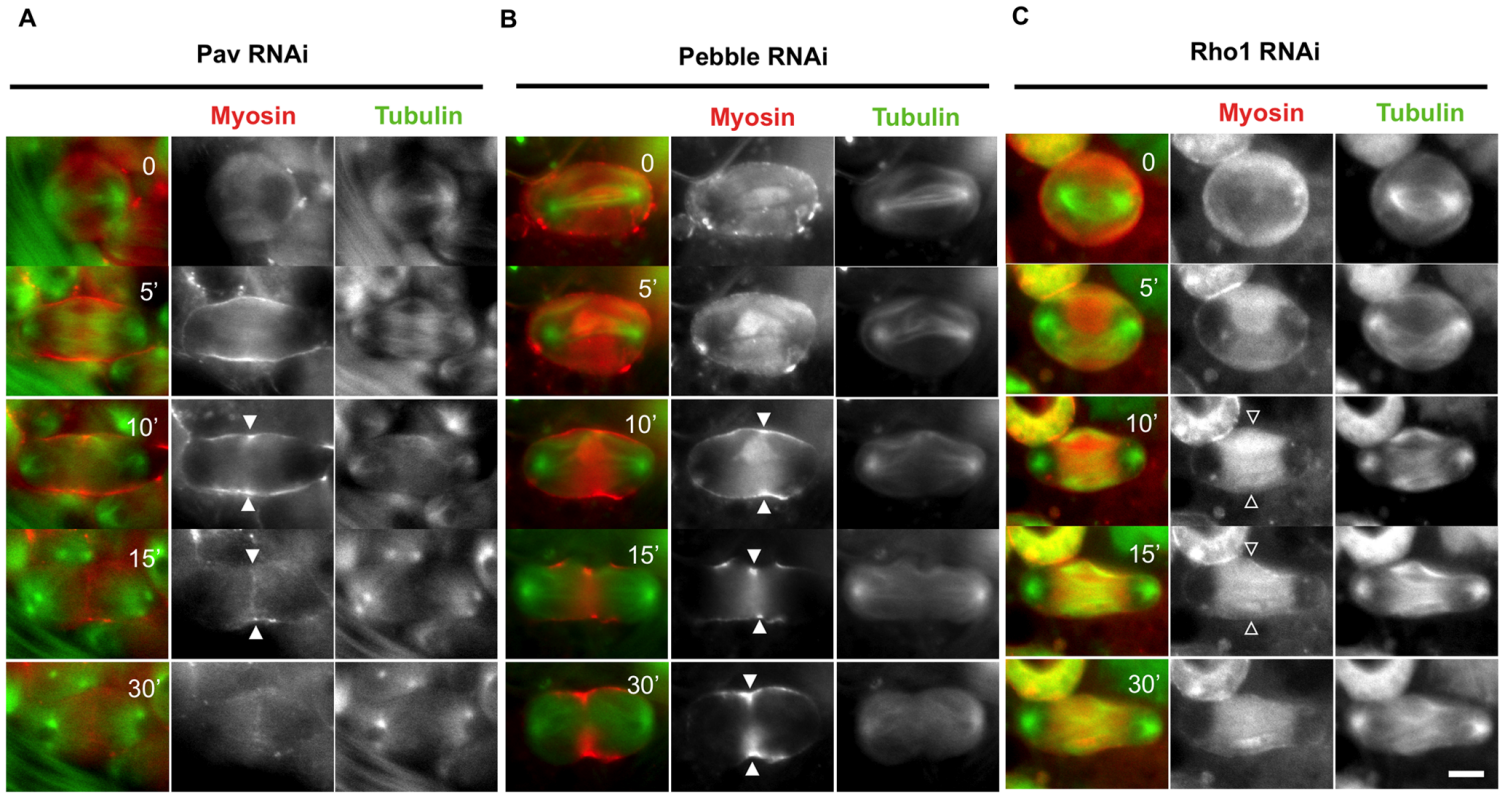
Accumulation of myosin II on the CF region is independent of centralspindlin. (A–C) Time-lapse observation of GFP-Sqh (red) with RFP-Tubulin (green) in primary spermatocytes. The images illustrate primary spermatocytes from the testis with simultaneous expression of dsRNA for *pav* (A), *pebble* (B), or *rho1* (C). Time in each panel is represented in minutes relative to the onset of anaphase I (t = 0 min). Filled arrowheads show the focus of myosin, while the open arrowheads indicate the absence of the focus. (A) Myosin accumulated as a ring on the CF region of a *pav*-depleted spermatocyte (myosin accumulated in 11/11 cells scored). (B) The myosin ring can be observed in the *pebble*-depleted spermatocyte (31/31 scored). (C) In the *rho1*-depleted spermatocyte, myosin accumulation could not be detected (1/26). Scale bar  = 10 µm.

We next examined whether centralspindlin consisting of RacGAP50C and Pav is required for myosin II accumulation at the presumptive furrow, at the CR and maintenance at the CR. These components accumulated in single rings in the CF region (Fig. S2A, B in File S1). In a control cell, a myosin ring was clearly formed in the CF zone. Surprisingly, the spermatocyte-specific expression of *pav* dsRNA did not affect myosin accumulation in the CF zone (Fig. 2A, Movie S4), although cytokinesis did not occur until the end of meiosis. These data indicate that myosin II accumulation in the presumptive CF zone of spermatocytes occurs independently of the centralspindlin complex. On the other hand, the myosin ring that was constructed at the mid-zone of the cell (arrowheads, t = 15 min, Fig. 2A) disappeared later (t = 30 min). Accordingly, CF ingression halted in the middle (t = 15 min) and ultimately regressed (t = 30 min). Thus, centralspindlin is required for the maintenance of myosin rather than for its accumulation at the CR in male meiosis.

We then examined whether Rho1 and its activator, Pebble, were required for these processes. It is known that Rho1 activation mediated by centralspindlin triggers F-actin polymerization during cytokinesis. Induction of dsRNA for Rho1 resulted in a complete failure of cytokinesis. We found that myosin II accumulation in the CF was entirely disrupted (Fig. 2C), although its initial recruitment along the cell cortex was observed (Fig. 2C, t = 0∼5 min). This clearly indicated that Rho1 is dispensable for initial myosin recruitment on the cell cortex but is required for its accumulation at the CF during late anaphase. Pebble, which is known as a local activator of Rho1, also accumulated in single rings in the CF region (Fig. S2C in File S1). Expression of the dsRNA successively provided effective depletion of the Pebble protein (Fig. S3C in File S1). The myosin accumulation in the CF zone was not affected in these *pebble*-depleted cells (arrowheads, Fig. 2B, Movie S5), whereas the already formed myosin ring began to disappear later (t = 30 min in Fig. 2B and thereafter; 31 out of 31 scored). These observations suggested that one of the RhoGEF proteins, Pebble, is dispensable for myosin accumulation in the CF zone and at the CR, whereas it is essential for myosin ring maintenance. Although the accumulation of the myosin ring was not affected, we observed that formation of defective central spindle microtubules was perturbed in the *pebble-*depleted cells (t = 15∼ min, Fig. S3B in File S1). Even when myosin rings were temporally assembled in *pav*-depleted or *pebble*-depleted cells, they disappeared shortly thereafter (arrowheads in Fig 2A and Fig. 2B, respectively). Centralspindlin and Pebble/RhoGEF are dispensable for myosin ring formation in the CF region, but they are required for maintenance of the myosin II ring.

### Orbit is required for myosin II accumulation at the equatorial cortex zone

**Figure 3:**
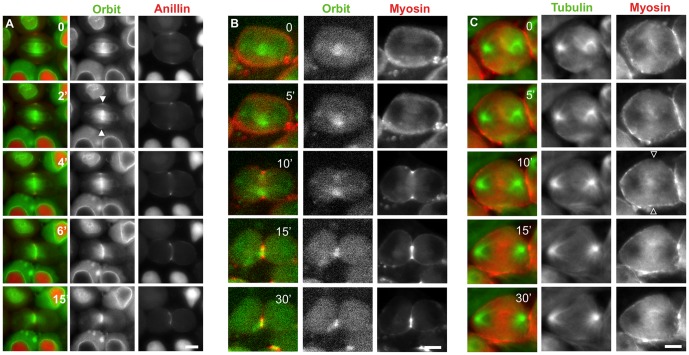
Orbit is required for myosin ring formation and its maintenance in the contractile ring. (A) Time-lapse observation of Orbit and anillin in spermatocytes with simultaneous expression of RFP-anillin (red) and GFP-Orbit (green). Time in each panel is shown in minutes relative to the onset of anaphase I (t = 0 min). Note that Orbit localization at the equatorial cortex (arrowheads) is coincident with anillin localization at the cortex. (B) A spermatocyte with the expression of RFP-Orbit (green) and GFP-Sqh (red). Orbit distribution partially overlaps with that of MLC and is distributed on the inside of the constricting myosin ring. (C) Myosin II dynamics (red) in spermatocytes of the hypomorphic *orbit* mutant. In a spermatocyte from the *orbit*7 mutant, MLC did not accumulate to the CF (open arrowheads) (This type of abnormal myosin distribution was observed in 3 of 8 cells examined). Green staining indicates RFP-tubulin.

**Figure 4:**
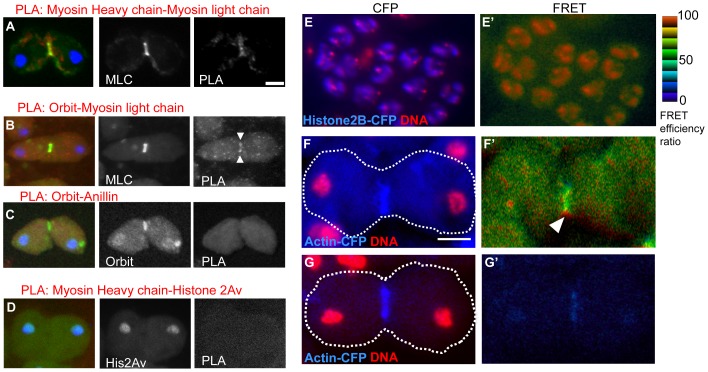
*In situ* PLA and FRET experiments suggesting a close association between Orbit and Myosin. (A–D) *In situ* PLA of late anaphase to telophase cells with expression of each GFP fusion expression. (A) Positive control for *In situ* PLA. The primary spermatocytes with GFP-sqh/MLC (green) were immunostained with anti-MHC antibody. PLA signals (red) confirmed a close (within 40 nm distance) association between MHC and MLC in the contractile ring (32 PLA positive cells/32 total telophase I cells scored). (B) The primary spermatocytes were immunostained with anti-Orbit antibody and anti-GFP antibody for GFP-sqh (green). PLA signals (red) also suggested a close association between Orbit and MLC, as distinctly shown in the contractile ring, whereas no PLA signal (red) for a close association between Orbit and anillin was observed in the ring at telophase (C) (0 PLA positive cells/more than 20 total telophase I cells scored). No PLA signal (red) was observed in more than 20 telophase cells expressing GFP-Orbit immunostained with anti-anillin and anti-GFP to detect GFP-Orbit (green). (D) Negative control for *In situ* PLA. The primary spermatocytes with GFP-Histone 2Av (green) were immunostained with anti-MHC antibody. No PLA signal (red) between the nuclear protein and the contractile ring protein was observed in more than 20 telophase cells. (E–G) FRET experiments to assess the close association between F-actin and Orbit. (E, E′) Positive control for the FRET experiment in early spermatocytes. Co-expression of CFP-histone 2B to YFP-histone 2B was induced using the UAS/Gal4 system. CFP-Histone 2B (blue) localized in nuclei in the spermatocytes; DNA (red). A FRET/CFP emission ratio was calculated using MetaMorph software. It is well known that histone H2B forms a dimer in a nucleosome, and therefore the YFP emission is generated because of FRET from CFP to YFP. The YFP-fluorescence generated by the FRET is clearly evident in nuclei (E′). (F, F′) CFP-actin (blue) localized on the CR at telophase I in spermatocytes. (F′) A Venus-fluorescence that would be generated by the FRET from the CFP to Venus is clearly evident on the contractile ring (arrowhead). (G, G′) Negative control for the FRET experiment. Co-expression of CFP-actin (G) and YFP-Asl was induced. (G′) No significant FRET signal between the contractile protein and the centriolar protein was observed in nuclei of more than 20 spermatocytes examined. The distribution of the ratio between the minimum value (0, darkest blue) and the maximum value (100, lightest red) is represented by the 8-step color indicator in the intensity-modulated display mode. FRET efficiency ratio = Venus fluorescence intensity ×100/CFP fluorescence intensity. Scale bars  = 10 µm.

A previous study showed that Orbit is essential for cytokinesis in male meiosis and that its hypomorphic mutation disrupted the formation of both anillin and F-actin rings [33]. Hence, we examined whether Orbit would be required for myosin accumulation in the prospective CF zone. Although previous immunostaining experiments have revealed that Orbit is localized in the interior CS, it was not clear whether Orbit is localized on the peripheral CS [33]. To examine the spatiotemporal localization of Orbit, we induced expression of GFP-Orbit or RFP-Orbit, both of which can rescue the male sterile phenotype of the *orbit* mutant [42]. We showed that their localization was consistent with the immunostaining results, except that faint but significant foci were temporally observed at the equatorial cortex (arrowheads, Fig. 3A). After the breakdown of the nuclear membrane, Orbit was concentrated in kinetochores (arrow, t = 0 min, Fig. 4A) and distributed along the spindle envelope (arrow, t = 5 min). It subsequently accumulated on the peripheral and interior central spindles toward the cell mid-zone at early anaphase (t = 5 min). Orbit was concentrated in the equatorial cortex, where peripheral MTs from opposite poles met at the plus-ends and overlapped (t = 10 min). The cortical Orbit foci on the prospective CF region coincided with the overlapping region of the peripheral MTs. Orbit was maintained in the CR during and after cytokinesis (t = 40 min).

We performed simultaneous observations of the localizations of Orbit, anillin (Fig. 3A), and myosin (Fig. 3B, Movie S3B). Orbit clearly accumulated in the CF zone as a ring (large arrowheads, Fig. 4A) and it was co-localized with the other two CR components. Orbit appeared to be concentrated in the inner position relative to myosin (Fig. 3B), while its foci appeared to be nearly overlapped with those of anillin (Fig. 4A). To examine close association between Orbit and CR components, we carried out the *in situ* proximity ligation assay (PLA). As a positive control, the *in situ* PLA signals between MHC and MLC were clearly found on the CR due to their binding (Fig. 3D). We confirmed the absence of the signals between MHC and Histone 2Av in telophase I spermatocytes as a negative control. We subsequently observed clear *in situ* PLA signals between Orbit and MHC were in close proximity (within 40 nm) in the CR (Fig. 4A) and in ring canals in the fixed spermatocytes (data not shown). However, we were unable to detect any *in situ* PLA signals between Orbit and anillin at the equatorial region or on the central spindles (Fig. 4B). Furthermore, we carried out a FRET experiment to confirm the close interaction between Orbit and F-actin. We initially illuminated the telophase I cells with co-expression of CFP-Histone 2B and YFP-Histone 2B at the CFP-excitation wavelength and we measured the generated fluorescence by FRET from CFP to YFP (Fig. 4E, E′). On the other hand, any FRET signal over detectable level was not observed in spermatocytes with co-expression of CFP-actin and a centrosome protein, YFP-Asl (Fig. 4G, G′). Subsequently, primary spermatocytes with co-expression of CFP-actin and Venus-Orbit (Fig. 4F) were illuminated at the CFP-excitation wavelength and measured Venus fluorescence. A considerable FRET signal was observed in the CF zone (Fig. 4F′). This result suggested that CFP-actin and Venus-Orbit were co-localized within a distance of 10 nm where effective FRET could occur. These data indicated that Orbit is closely associated with MHC or F-actin in the CR. This result is consistent with our previous FRET results, which suggested a close association between Venus-Orbit and CFP-actin on the fusome of premeiotic spermatocytes [42].

Inoue et al. [33] showed that neither anillin nor F-actin was localized properly in the CF in *orbit* ***^7^*** mutant spermatocytes, suggesting that accumulation and/or maintenance of both anillin and F-actin requires Orbit. We examined the localization of myosin subunits in spermatocytes from hypomorphic *orbit* ***^7^*** mutants using anti-Zip antibody. The *orbit* mutation could not stabilize the peripheral MTs, and no MHC was detected in the mid-zone of telophase-like cells from the *orbit* mutant spermatocytes (data not shown).

Furthermore, we examined myosin dynamics in living spermatocytes from the mutant (Fig. 3C, Movie S7, Fig. S4 in File S1). Some late anaphase-like spermatocytes from hypomorphic *orbit* mutants did not display myosin II accumulation at the CF region, in which peripheral MT bundles failed to construct (3 out of 8 primary spermatocytes examined; Fig. 3C). Such abnormal mutant cells, without stable peripheral MT bundles, failed to initiate furrowing, although they displayed initial myosin recruitment. Orbit accumulation on the cortical region or at the mid-zone of the CS failed to appear until the end of meiosis I. In other hypomorphic mutant cells, myosin recruitment on cortex as well as accumulation on the CF occurred properly (5 out of 8 spermatocytes examined; Fig. S4 in File S1). However, the myosin ring collapsed at the later meiotic stage. Furrow ingression was initiated, but soon after, it was abruptly arrested and ultimately regressed. In these types of cells, peripheral MT bundling and subsequent myosin ring accumulation initially occurred properly and the furrowing was initiated. Thereafter, however, the myosin ring on the CR ultimately collapsed. This observation is consistent with previous results showing that the *orbit* mutation influenced the formation of the peripheral CS and resulted in the disruption of myosin ring formation at the cell mid-zone [33]. In summary, Orbit is required for myosin accumulation at the equatorial cortex for the construction and stabilization of the peripheral CS, and is also required for the maintenance of myosin ring to facilitate CF ingression.

Furthermore, we carried out genetic experiments to confirm the interaction between Orbit and myosin during cytokinesis. A failure of cytokinesis in meiotic division was detected based on the presence of multinucleated spermatids [33], [43]. The hypomorphic mutant *orbit* ***^7^*** displayed cytokinesis defects in male meiotic divisions at a low frequency (Table 1, [33]). The frequency of multinucleate spermatids increased significantly after introducing a single copy of an amorphic allele for *zip* (Table 1). This genetic data implied a close interaction between Orbit and myosin in male meiotic cytokinesis.

**Table 1:** Genetic interaction between *orbit* and *zip* in male meiotic cytokinesis appeared in onion stage spermatids.

Genotype	Total cells scored	Nebenkern-to-nuclei ratio (cells)								macro/micronuclei (cells)
		Normal	Abnormal							
			1/0	1/1	1/2	1/3	1/4	1>5	N	
*orbit^7^/+*	1124	100	0	0	0	0	0	0	0	0
*orbit^7^*	960	94.1	1.3	1.1	0.4	0	3.1	0	5.9	1.8
*zip^1^/+*	1016	100	0	0	0	0	0	0	0	0
*zip^1^/+;orbit^7^/+*	979	100	0	0	0	0	0	0	0	0
*zip^1^/+;orbit^7^*	356	52.8	7.0	8.1	9.6	3.1	14.6	4.8	47.2	30.9

### Orbit accumulation at the cell equatorial zone is dependent on MTs but not on F-actin or myosin II, whereas its maintenance requires these CR components

**Figure 5:**
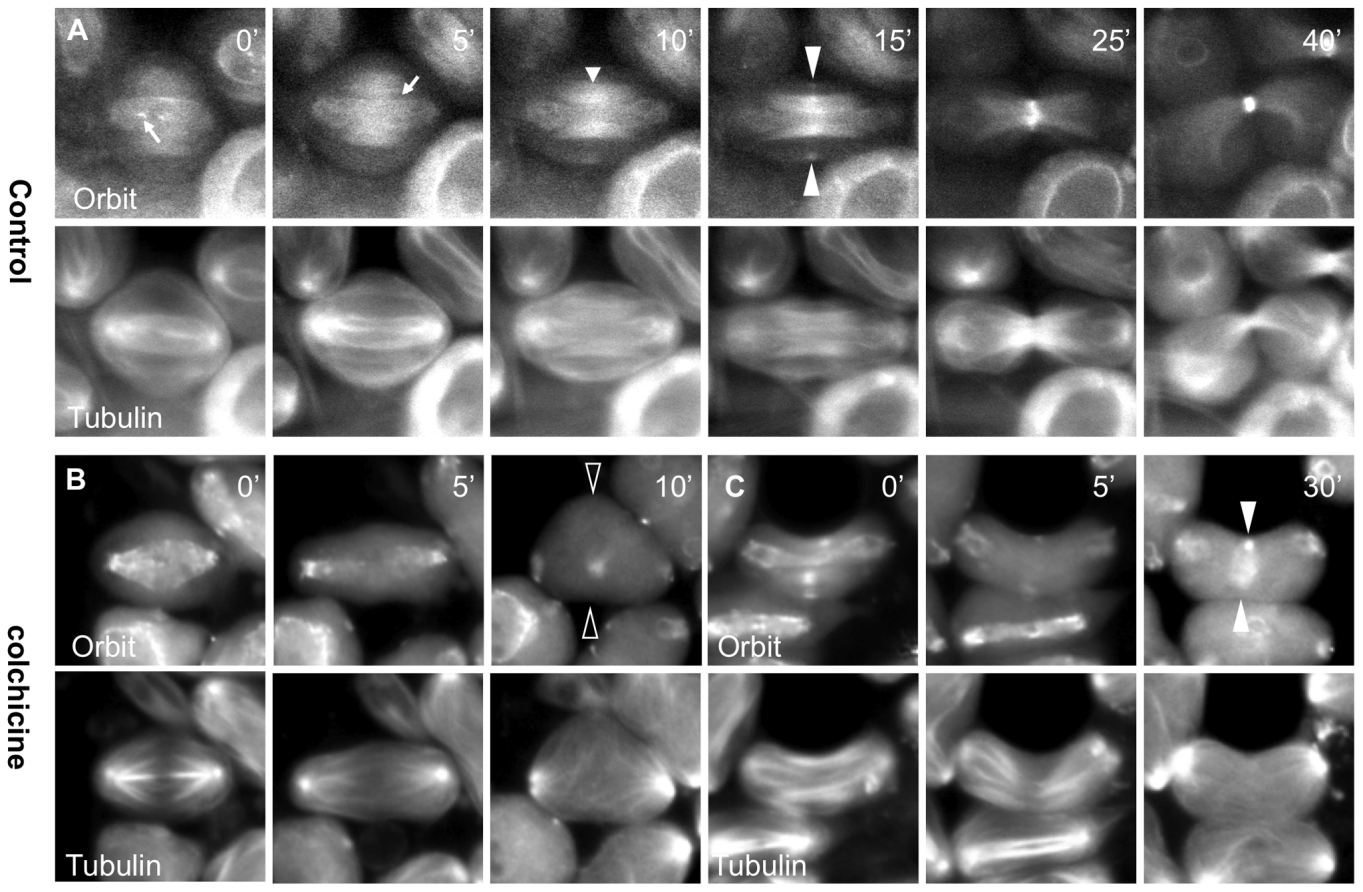
MTs are required for Orbit accumulation on the CF. (A) Time-lapse observation of Orbit and microtubules in spermatocytes expressing GFP-Orbit and RFP-tubulin undergoing meiosis I. After a nuclear membrane breakdown, Orbit is concentrated on the kinetochore (arrow, t = 0 min) and spindle envelope (arrow, t = 5 min). It then begins to accumulate at the cell mid-zone (t = 5 min). Orbit is more concentrated at the central region where peripheral MTs from opposite poles overlap (small arrowhead, t = 10 min). The cortical Orbit foci on the prospective CF region coincide with the overlapping region of peripheral MTs (large arrowheads, t = 15 min). After cytokinesis initiates, the propagation of CF compresses both peripheral and interior MT bundles into a common central spindle. (B) Time-lapse observation of Orbit and microtubules in spermatocytes under the presence of colchicine. Note that the spindle microtubules become degraded in this condition. Most of the cortical Orbit foci were not observed (open arrowheads, 0 cells with Orbit foci of the 21 cells examined). (C) However, living spermatocytes in which the Orbit rings had already been constructed before colchicine addition maintained the foci in spite of the absence of cortical MTs (filled arrowheads) (16/17). Scale bars  = 10 µm.

**Figure 6:**
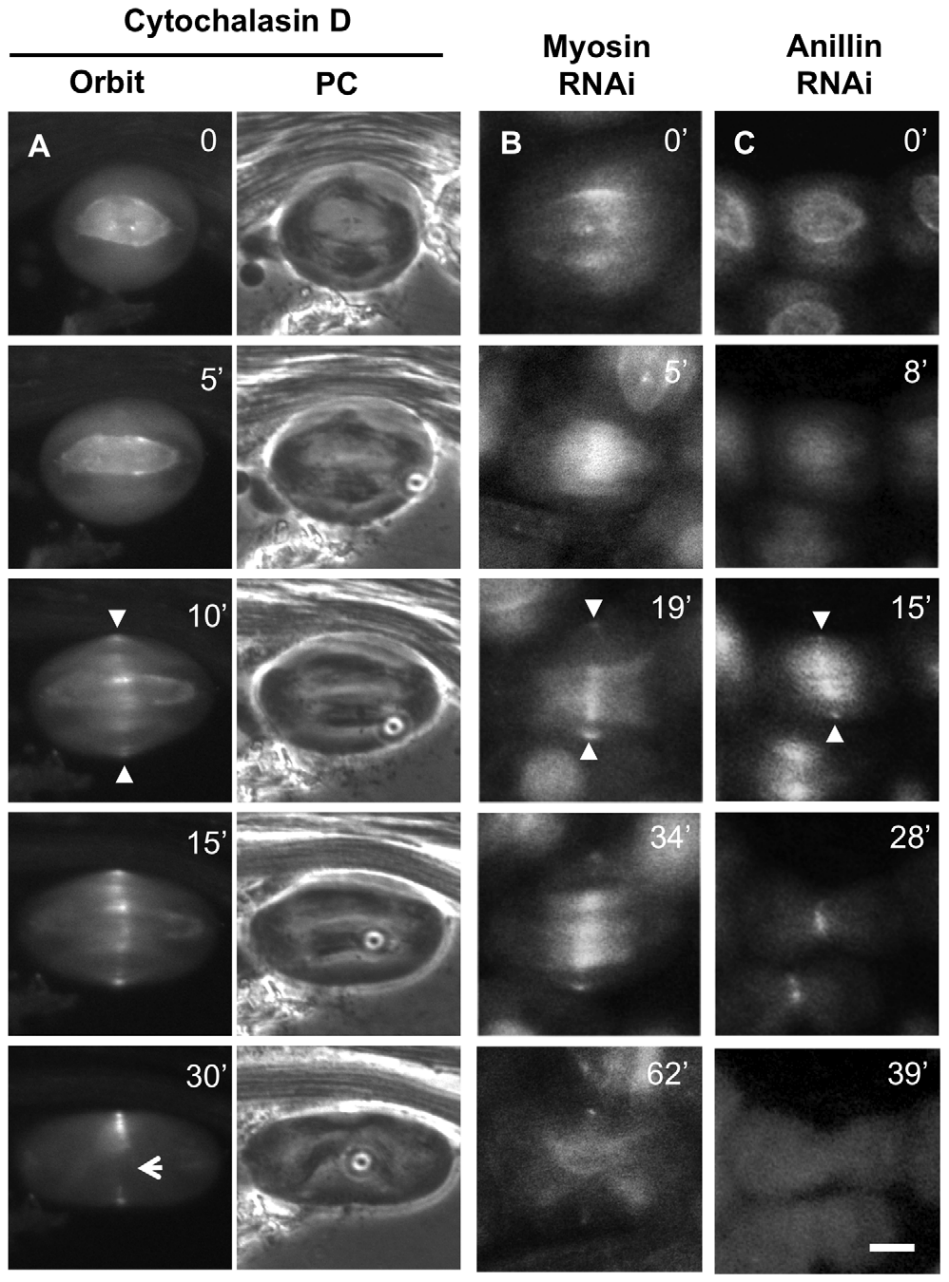
Accumulation of Orbit in the CF region is independent of F-actin, myosin, or anillin. Time-lapse observation of Orbit in meiosis I. (A) A wild-type spermatocyte treated with cytochalasin D. The Orbit foci on the CF (arrowheads) can be observed in the treated cell (15 cells had Orbit foci on the CF/19 cells examined). Most of the foci appeared temporally and faded out later (arrow, t = 30 min). (B) Depletion of *zip*. Note that Orbit accumulation was observed on the CF in 15 of the 15 cells examined arrowheads). (C) Depletion of *anillin*. Cortical Orbit foci were detected in 20 of the 23 cells examined (arrowheads). Timing of anaphase onset (t = 0 min). The filled arrowheads show the presence of Orbit accumulation. Scale bars  = 10 µm.

To identify the factors required for the localization of Orbit in the equatorial zone cortex, we initially examined whether this localization was dependent on MTs (Figs. 5B, C), F-actin (Fig. 6A), myosin (Fig. 6B, Movie S8), or anillin (Fig. 6C, Movie S9). We treated normal live spermatocytes with colchicine and simultaneously observed Orbit and MTs (Fig. 5B, C). Spindle MTs were clearly degraded, and few cortical Orbit foci were observed [e.g., comparing treated cells (Fig. 5B at t = 10 min) to a normal cell (Fig. 5A at t = 10 min)]. However, if colchicine was added to cells in which Orbit foci had already formed, the foci were maintained without cortical MTs (arrowheads in Fig. 5C). Next, we examined whether Orbit accumulation in the CF region and its maintenance were influenced by F-actin (Fig. 6A). In the presence of cytochalasin D, we observed that Orbit could assemble in a ring at the equatorial cortex (arrowheads in Fig. 6A), while the Orbit band at mid-zone of the cell disappeared without distinct furrowing (arrow, t = 30 min, Fig. 6A). These data suggest that the accumulation of Orbit in the cortex is dispensable for F-actin, although this cytoskeleton plays a role in its maintenance. Furthermore, the *MHC*-depletion did not show any effects on the formation of Orbit foci in the equatorial cortex (Fig. 6B). Orbit accumulated normally in the cortex (arrowheads in Fig. 6B), although it failed to accumulate as a single band (t = 15 min, Fig. 6B). Orbit accumulation in the CF did not require myosin, whereas the accumulation and maintenance of the myosin ring depended on Orbit. This is consistent with previous reports that indicated that F-actin is required to stably maintain myosin II in the CR. Expression of dsRNA for *anillin* successfully depleted the protein from cells (data not shown). In the anillin-depleted cells, the Orbit ring was initially constructed but then ultimately disappeared from the CR region (Fig. 6C). In summary, Orbit ring formation in the prospective CF zone depends on MTs but not on myosin II, F-actin, or anillin. After the accumulation of the Orbit ring was completed, F-actin, myosin, and anillin influenced the maintenance of the Orbit ring, which was not dependent on the MTs.

### The KLP3A/Feo complex is required for Orbit accumulation in the CF zone

**Figure 7:**
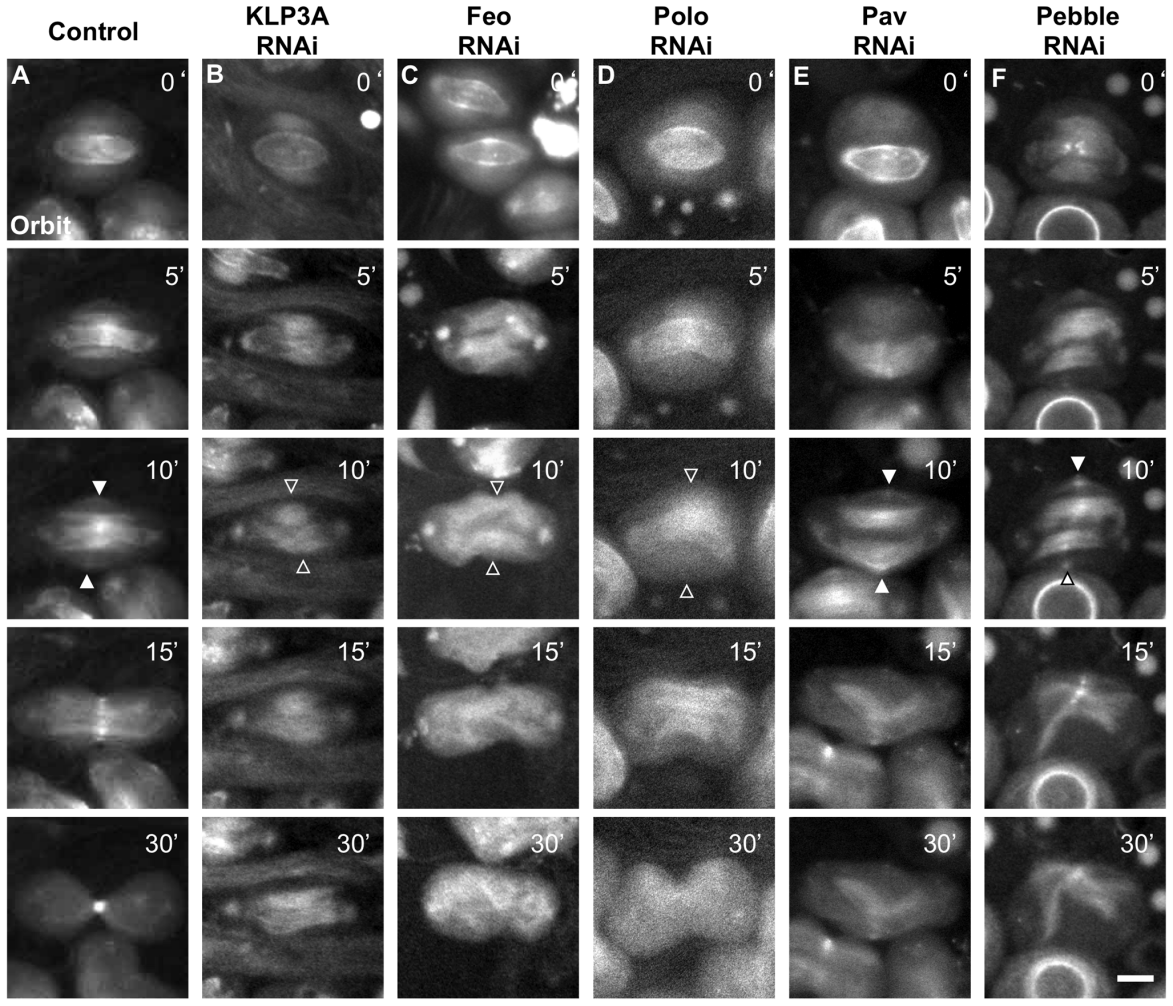
The Orbit accumulation on the CF depends on KLP3A, Feo and Polo. Time-lapse observation of Orbit in spermatocytes expressing dsRNA for 6 cytokinesis-related genes. Filled arrowheads show the focus of myosin, while the open arrowheads indicate the absence of the focus. (A)Normal control. (B)Depletion of *KLP3A*. Orbit foci on the CF were not detected (0 cells with detectable Orbit foci on the CF/18 total cells examined). (C) Depletion of *feo*. The Orbit foci were not observed (0/11). (D) Depletion of *polo*. The Orbit foci were not observed (0/5). (E) Depletion of *pav*. The Orbit foci appeared on the CF (10/12). (F) Depletion of *pebble*. The foci were observed on the CF (5/5). Time of onset of CF ingression was set at t = 0 min. Scale bars  = 10 µm.

To identify other essential factors for Orbit accumulation in the CF zone, we first carried out an RNAi screening designed to identify the genes required for cytokinesis in spermatocytes. We selected 86 candidate genes related to cytokinesis and analyzed them using 179 RNAi stocks (see Materials and Methods). From the results of spermatocyte-specific expression of dsRNA, we identified 22 genes that are known to influence cytokinesis of male meiotic division (Table S1). We then examined whether Orbit foci were affected in spermatocytes of F_1_ males derived from crosses between the 179 RNAi stocks and the *bam-Gal4* stock by using time-lapse observations. Consequently, we found three genes, *klp3A, feo*, and *polo*, which influenced the Orbit localization (Figs. 7A–D, Movies S10, S11). As KLP3A and Feo form a complex required for the transport of Polo to the CF in S2 cells [44], our results suggested that the accumulation of Orbit requires targeting of Polo at the CF by using the KLP3A MT motor and Feo.

Next, we examined whether Orbit accumulation in the CF zone depends on centralspindlin. Pav localization overlapped with that of Orbit through most of meiosis (Fig. S2A in File S1). However, we never detected *in situ* PLA signals suggesting direct binding between Pav and Orbit. We observed Orbit foci in the equatorial cortex in *pav*-depleted cells during late anaphase, although they appeared temporarily and quickly vanished (arrowheads in Fig. 7E). These observations suggested that transport of Orbit toward the CF zone was independent of Pav kinesin-like motor protein. Moreover, depletion of *pebble* did not affect Orbit accumulation, indicating that Pebble is also dispensable for this process. In addition, we investigated the requirement of Rho1 for Orbit ring formation. Rho1 displayed different cellular localization in spermatocytes than it did in S2 cells. In male meiosis, Rho1 was associated with an ER-based structure known as the spindle envelope at anaphase (Fig. S2D in File S1). Depletion of *rho1* did not entirely affect Orbit accumulation, although cytokinesis did not occur in the depleted spermatocytes (data not shown). Our data indicated that Orbit accumulation in the CF zone was independent of the centralspindlin complex and Rho1.

### Mutations of consensus sequences for phosphorylation by Cdk1 or Polo in Orbit did not affect Orbit localization in the CF region

**Figure 8:**
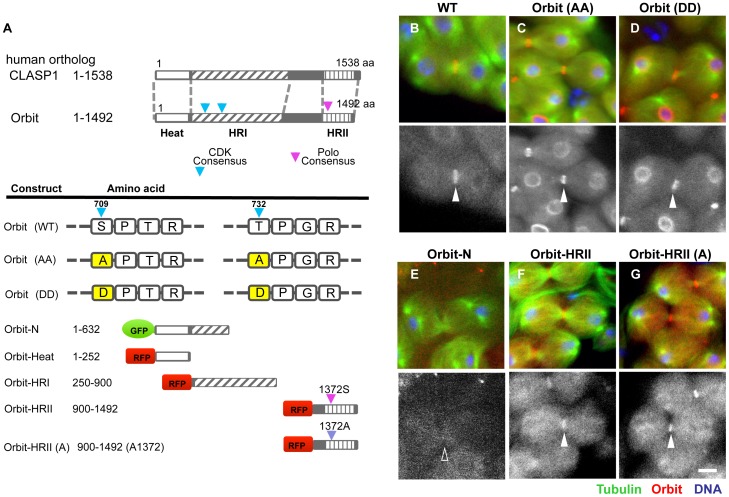
The C-terminal conserved region, HR II, of Orbit is sufficient for its localization on the CF region. (A) Schematic representation of the conserved Orbit region and the surrounding sequences that match the consensus target site of Cdk1/Cyclin B (blue arrows) or Polo kinase (pink arrows). (B) Control telophase I spermatocyte with GFP-Orbit (red). RFP-Tubulin (green). (C, D) Spermatocytes with expression of amino acid substitution mutants of possible phosphorylation sites by Cdk1/Cyclin B (S709 and T732). (C) Ala substitution mutant (indicated as Orbit(AA), red) in possible Cdk1 phosphorylation sites. RFP-tubulin (green). (D) Asp substitution mutant (indicated as DD, red). RFP-tubulin (green). A single representation of Orbit localization (lower panels). Both mutant proteins showed normal localization at the CR (red). (E) Absence of localization of Orbit-N region (1–632) (red) at the CF zone in telophase I spermatocyte. RFP-tubulin (green). Both mutant proteins showed normal localization at the CF zone. (F) Expression of the HR II region (250–900) without mutation (S1372). Upper panels (GFP-tubulin, green), (RFP-HR II, red). lower panels (RFP-HR II). Note that the HR II region alone is sufficient for its localization on the CF. (G) Expression of the HR II region carrying the A1372 mutation. Its normal localization at the CF region and the CR were observed. Scale bars  = 10 µm.

To clarify the regulatory mechanism that facilitates the localization of Orbit in the CF region, we first determined the Orbit domain sufficient for its localization to the CF. We induced expression of highly conserved regions within Orbit: Heat (1–252 amino acid residues), N-terminal region (1–632), HRII (250–900), and HRII (900–1492) (Fig. 8A, [36]). Inoue et al. reported that the N-terminal region possessed MT binding activity in cell extracts. However, with the exception of HRII, none of these polypeptides displayed significant localization on peripheral MTs or accumulation in the CF zone (Fig. 8E, F). Surprisingly, HRII alone was sufficient for the localization of the CF and the CR, but not the MT structures, during meiosis. These data suggest that Orbit is localized in the CF through the HR II region independently of MTs.

We substituted the amino acid residues in the consensus sequences for phosphorylation by Cdk1 and Polo kinases. We first replaced both serine^709^ and threonine^732^ residues in the consensus sequences for Cdk1 with alanines or asparagines, which mimic constitutive phosphorylation and dephosphorylation, respectively (Miyauchi and Inoue, unpublished). However, both mutant proteins, OrbitD^709^D^732^ and OrbitA^709^A^732^, displayed indistinguishable localization from the control protein (Fig. 8B–D).

The amino acid sequence DLS^1372^I is present in the HRII region, which matches the consensus sequence for Polo phosphorylation sites (see Materials and Methods). Therefore, we substituted the serine^1372^ residue to alanine by *in vitro* mutagenesis and examined the cellular localization of the mutant polypeptides in male meiosis. The mutant HRII protein also displayed localization that was indistinguishable from that of the control protein (Fig. 8F, G).

## Discussion

In order to understand the molecular mechanism of CR formation in cytokinesis, we examined the assembly of each CR component to the prospective CF region by time-lapse observation of *Drosophila* spermatocytes. We found that myosin II was initially recruited on the cell cortex independently of MTs. Then, peripheral MTs attached to the equatorial cortex, serving as a cue to initiate CR formation. Shortly after, myosin II was concentrated in a ring on the equatorial cortex dependent on MTs. On the other hand, anillin and F-actin assembled abruptly in a ring on the equatorial cortex where the MTs made contact, simultaneously with myosin II accumulation in the CF region. Furthermore, both centralspindlin and Pebble/RhoGEF were dispensable for the myosin II recruitment and its accumulation at the CF region. Conversely, they were indispensable for maintenance of the ring. Although Rho1-depleted spermatocytes maintained cortical recruitment of myosin, Rho1 was indispensable for myosin II accumulation. In order to identify the regulatory factors involved in the recruitment of the CR proteins, we examined the cellular localization of Orbit, which is a *Drosophila* ortholog of a microtubule-associated protein, CLASP. Orbit was localized at the mid-zone of cortical MT bundles and shifted toward the prospective CF site at late anaphase. Our PLA *in situ* and FRET experiments showed that Orbit was closely associated with both F-actin and myosin II in the CR. We showed that myosin II accumulation required Orbit. However, centralspindlin was dispensable for Orbit accumulation. Instead, Polo and the KLP3A-Feo complex, which is essential for Polo recruitment, were required for Orbit accumulation independent of its MT-binding domain. A requirement of cytoskeletal proteins, contractile ring proteins and their regulatory proteins for each step of myosin II localization in cytokinesis of meiotic divisions was summarized in Table S2. However, mutations of the consensus amino acid sequences for phosphorylation by Cdk1 or Polo did not influence its cortical accumulation. Orbit is also necessary for maintenance of the CR through a close association with myosin and F-actin in the CR. Based on these results, we speculate that Orbit plays an essential role as a connector between MTs and the CR during cytokinesis in *Drosophila* male meiosis.

### Myosin ring formation at the CF site is divided into two sequential steps regulated by different mechanisms

Our observations of living spermatocytes showed that the accumulation of a myosin subunit at the prospective CF region could be divided into two successive steps: recruitment along the cell cortex and accumulation to the equatorial cortex region. Two other components, F-actin and anillin, abruptly appeared in a ring on the restricted cortex region where the peripheral MTs made contact. In both *Drosophila* larval neuroblasts and embryonic cells in *Caenorhabditis elegans*, it was reported that myosin accumulation in the CF region occurs in two steps; the first involves recruitment of the myosin subunit to a wide region along the cortex [45], [46], and the second involves restricting its wide distribution in the equatorial region. Uehara and coauthors (2010) also described the two-step accumulation of myosin II in *Drosophila* S2 cells [47]. In contrast, Giansanti and Fuller (2012) suggested that spermatocytes appear to skip the first step of myosin accumulation [6]. However, our analysis in the presence of colchicine indicated that myosin accumulation at the CF site was divided into two steps; the first step (recruitment) was independent of MTs, and the next step (restriction) was dependent on MT structures. As these two successive steps may proceed continuously under normal conditions, it may be difficult to recognize the processes as separate phases without careful observation of living cells. Furthermore, it has also been reported that the initial recruitment is performed dependent on Rho during early anaphase of S2 cells [48], [47]. However, we observed myosin recruitment along the cell cortex in *rho1*-depleted spermatocytes, although subsequent accumulation of the cortical myosin was entirely inhibited. As we did not succeed in detecting the accumulation of GFP-Rho1 at the CF site (Fig. S2D in File S1), its involvement in myosin recruitment in this cell type should be interpreted with caution. In the second step, the initial distribution of myosin is subsequently narrowed to the equatorial cortex zone, depending on CS MTs. A previous study demonstrated that peripheral MTs from opposite spindle poles meet at the mid-zone and make contact with the cortex in spermatocytes [33]. We speculate that this MT attachment may serve as a cue to initiate myosin accumulation. This is consistent with observations that the assembled myosin filaments were found to be dependent on MTs in S2 cells [47], [49].

In contrast to the two-step accumulation of myosin, anillin or F-actin appeared abruptly as a narrow band in spermatocytes. Recruitment and assembly of anillin and F-actin may occur continuously in response to MT attachment in spermatocytes. In the embryonic cells of *C. elegans*, anillin localization appeared to occur in two steps, similar to that of myosin [46]. Therefore, the regulatory mechanism to recruit the CR components may differ among different cell types or organisms.

### Centralspindlin is dispensable for recruitment of the CR components but is crucial for CR maintenance in *Drosophila* spermatocytes

It is generally believed that centralspindlin provides local activation of Rho, and that it initiates CR formation at the equatorial cortex in animal cells [23]. Studies in *Drosophila* and mammalian cells indicated that the activation of Rho1 at the CF site requires Pebble/RhoGEF, which is a Rho1 activator [50], [51]. It was also shown that the localization of the GAP component, RacGap50C, in the centralspindlin complex was sufficient for the local activation of Rho1 in *Drosophila* S2 cells [52]. We showed that the initial recruitment or equatorial accumulation of myosin II in the CF region did not require centralspindlin. This was consistent with previous results indicating that the initial localization of myosin II did not require centralspindlin, but was instead dependent on Pebble, Rho1, and Rho kinase in S2 cells [48]. The myosin accumulation at the prospective CF site occurred soon after the attachment of peripheral MTs to the cortex (Fig. 1C). D'Avino and coauthors (2006) observed that astral microtubules serve to deliver the centralspindlin complex to the equatorial cortex [52]. The attachment of peripheral MTs to the cortex may convey the Rho1 activator to the prospective CF site. Curiously, we observed normal localization of myosin as a narrow band in *pebble*-depleted spermatocytes, although we confirmed that its dsRNA expression was sufficient for the 90% elimination of Pebble protein. This observation is inconsistent with results obtained from S2 cultured cells [48], [47]. We could not exclude a possibility that a remaining amount of Pebble is enough for myosin II accumulation at CF site but not for its maintenance in the CR. However, it is more likely to consider that two other RhoGEF proteins, RhoGEF2 and RhoGEF3 (http://flybase.org/), which were enriched in spermatocytes, could compensate for the *pebble* depletion.

We showed that the depletion of components in the centralspindlin resulted in regression of furrowing at the CF site. Consistent with this observation, the CR components assembled initially but soon disappeared from the CF site in the depleted spermatocytes. This was also consistent with the results obtained from S2 cultured cells [48]. The centralspindlin is therefore considered to be required for the maintenance rather than the formation of the CR.

### Orbit is crucial for myosin II ring formation and for CR maintenance by facilitating the connection between CS MTs and the CR in *Drosophila* spermatocytes

We attempted to elucidate the factors that allow myosin subunits to localize in the CF zone. Mutant spermatocytes for *orbit*, encoding a *Drosophila* ortholog of CLASP, failed to undergo cytokinesis during meiotic divisions [33]. We here showed that Orbit is closely associated with both F-actin and MHC in the CR. The protein is localized on the CS mid-zone during late anaphase, and is associated with a subset of MT bundles known as interior CS residing inside the nuclear envelope. In addition, our observations of living spermatocytes revealed a novel localization of Orbit in an overlapping zone of anti-parallel MTs below the cell cortex. Our previous study described that bundling of peripheral MTs was also severely affected in some cells without furrow ingression [33]. Our current observations demonstrated that localization of Orbit on peripheral MTs is a consistent phenomenon that enables the loss of MTs that was demonstrated in a previous study.

Although our results indicated that Orbit is required for the recruitment of myosin subunits to the prospective CF region, the mechanism by which the Orbit protein assembles in the CF region needs to be clarified. As anillin is also involved in MT bundling [26], it is reasonable to consider that anillin may be involved in the formation of the MT structure and is essential for the transport of Orbit to the CF site. The cortical localization of Orbit may be mediated through its direct binding to myosin, but not to F-actin, in the CR, because the distribution of Orbit was not perturbed by cytochalasin D.

The hypomorphic *polo* mutant failed to undergo cytokinesis in male meiosis [21], suggesting that Polo is required for the initiation and/or progression of cytokinesis in spermatocytes. We here showed that Orbit accumulation in the CF required Polo and the KLP3-Feo complex. However, *orbit* mutations at consensus phosphorylation sites for Polo and Cdk1 had no effect on recruitment to the CF region. We cannot exclude a possibility that the non-phosphorylatable mutant forms could make a functional complex with endogenous Orbit and it could be localized properly. Alternatively, Polo possibly may have indirect effects on Orbit recruitment, although biochemical analysis to examine whether the protein is phosphorylated by Cdk1 or Polo should to be performed in a future work. Polo interacts with centralspindlin by binding with RacGAP50C, and it triggers centralspindlin localization on the CF site [53]. It can be speculated that Orbit is associated with the complex and is conveyed to the site. Alternatively, Polo may interact with a plus-ended motor such as KLP61F. Orbit might be concentrated around the plus-ends of peripheral MTs by a plus-ended motor. There is evidence that mammalian Plk1 modifies the activity of mammalian kinesin-5 [54]. It would be particularly interesting to show that phosphorylated KLP61F could interact with Orbit and convey it to the CF site. We showed that Feo, a *Drosophila* ortholog of PRC1, is also required for Orbit recruitment. Feo is indispensable for the formation of CS MTs and cytokinesis in spermatocytes [34]. It was further reported that mammalian PRC1 is essential for polarizing antiparallel MTs and for concentrating factors for CR assembly [55]. It would be of interest to examine whether Orbit interacts with Feo in CR formation in spermatocytes.

On the other hand, CF formation and ingression in *Drosophila* cultured cells were unaffected by the depletion of Feo, which is essential for the recruitment of Polo to the spindle mid-zone [44]. The indispensability of Polo in cytokinesis differs between cultured cells and spermatocytes. Polo and the KLP3A/Feo complex are particularly important for cytokinesis in spermatocytes. The cell-specific role of the Polo/KLP3A/Feo complex may result from specific characteristics of cytokinesis in germline cells, which are interconnected by cytoplasmic bridges generated from the stabilization of the CR. As both Polo and Orbit continue to be localized on the bridges known as the ring canal, spermatocytes might need this additional regulatory system to stabilize the CR in addition to centralspindlin.

Inoue and colleagues (2004) reported that the CF ingression of *orbit* mutant spermatocytes was arrested, and that regression of furrowing occurred at the end [33]. These findings suggest that Orbit also plays an essential role in the later stages of cytokinesis. In the mutant spermatocytes, disintegrated anillin rings and a lack of F-actin rings were described. Since the fixed cells were observed after immunostaining, it could not be distinguished whether these abnormal cells were generated from the failure of CR formation or from collapse of the assembled CR. It is also important to clarify whether Orbit is required for the maintenance of the CR components. Anillin is necessary for the maintenance of actomyosin at the equator in late stages of cytokinesis in *Drosophila* spermatocytes [32]. In S2 cells, an interaction between anillin and RacGAP50C is required for maintenance of the connection between the actomyosin ring and spindle MTs [27]. Anillin can bind myosin and regulate CR activity [26]. Although we were not able to detect the direct interaction between Orbit and anillin, Orbit displayed co-localization with anillin. Orbit may strengthen the CR from the inside by connecting CS MTs and CR on the cell cortex. We also showed that Pav and Pebble are required for the maintenance of the myosin ring. Giansanti and coauthors proposed that the CS MTs interact cooperatively with the CR in cytokinesis during male meiosis. The molecular mechanism that regulates the linkage between these two cytoskeleton structures is not yet fully understood. Orbit is also required for the formation and maintenance of peripheral CS MTs [33]. In this study, we showed that Orbit is a novel regulator that is indispensable for the recruitment and/or formation of CR components in male meiosis. It also plays an essential role in the maintenance of the CR by possibly binding to MTs, F-actin, and myosin. Considering this evidence, we propose that the Orbit protein plays a role as a connector between CS MTs and the CR in cytokinesis.

## Materials and Methods

### *Drosophila* Stocks

*y w*, or *GFP-β-tubulin* stock [29] were used as a normal control for cytological and time-lapse studies. *GFP-Orbit*, *RFP-Orbit*, *Venus-Orbit*, *GFP-Orbit-N* (aa1-632), *RFP-Orbit-Heat* (1–252), *RFP-Orbit-HRI* (900–1492), and *RFP-Orbit-HRII* (250–900) stocks were described elsewhere [42]. We selected 86 candidate genes (179 RNAi stocks), including genes essential for cytokinesis in S2 cells, genes for all MT-based motors, and genes that showed genetic interaction with *orbit* in eye discs [56], [57], [58], [59]. The stocks containing *UAS-RNAi* constructs for genes related to cytokinesis were obtained from the Vienna *Drosophila* RNAi Center, Bloomington Stock Center, and National Institute of Genetics (Table S1). We used *bam-Gal4::vp16* [60] and *UAS-dcr2;bam-GAL4::vp16* (a gift from T. Noguchi) as a Gal4 driver for induced expression of spermatocyte-specific genes or of their dsRNA for knockdown experiments, respectively. The following stocks were distributed from Bloomington Stock Center: *RFP-Actin5C*, *GFP-Actin5C*, *CFP-Actin5C*, and *GFP-Rho1*. *GFP-anillin* and *RFP-anillin* were a gift from J.A. Brill. *GFP-Sqh* was obtained from R. Karess. *GFP-Pav* and *GFP-Polo* were from D. Glover. *GFP-RacGAP50C* was from S. Gregory [61]. *orbit^7^* was described previously [33]. *RFP-β-tubulin* was constructed by insertion of the α-tubulin cDNA into the pURW vector [62] using the Gateway recombination protocol (Invitrogen).

### *In vitro* mutagenesis of putative phosphorylation sites for Polo in Orbit

The various consensus sequences recognized by Polo kinase are as follows. In one sequence, D/E-X-S/T-Φ-X-D/E, the acidic D/E amino acids are preferred at positions −2 and +3, and a hydrophobic amino acid is preferred at the +1 position [63]. An alternative consensus sequence for Plk phosphorylation was proposed as Q/D/E-X-S/T-Φ, in which there is no specific requirement at position +3 [64], [65]. The flanking sequences surrounding 1370aa–1373aa in *Drosophila* Orbit conform to the Q/D/E-X-S/T-Φ consensus. The mutant sequence generated by substitution of serine for the wild-type alanine at position 1372 of Orbit conforms exactly to the consensus motif for phosphorylation by Polo kinase, D/E-X-S/T-Φ-X-D/E, with Φ indicating hydrophobic residue [64], [65]. This raises the possibility that phosphorylation of the mutant Ser-1372 by Polo might cause a conformation change that could affect Orbit activity during male meiosis.

### PLA method

*In situ* PLA is a method that enables detection of protein interaction with high specificity and sensitivity. *In situ* PLA methods were performed according to the Duolink kit method (Nacalai Inc., Kyoto, Japan). We applied the *in situ* PLA with a combination of antibodies that allowed the detection of binding between Orbit and MHC. We used anti-Zip to recognize MHC, and anti-GFP for GFP-Orbit detection. We repeated the experiment using Orbit antibody and anti-GFP for GFP-Zip detection.

### Live cell imaging of primary spermatocytes under drug treatments

We modified a protocol described in Inoue et al., 2004. Testes from adult flies were dissected and spread under mineral oil (Trinity Biotech plc.) on clean glass cover slips in open chambers surrounded by double-faced tape. Using this protocol, we have achieved continuous observation of primary spermatocytes from prophase I through a minimum of 1 hour of meiosis I. Time-lapse imaging was performed using an Olympus IX81 fluorescence microscope (Olympus, Inc., Tokyo, Japan) equipped with excitation and emission filter wheels (Olympus, Inc.). For each 30-sec interval, GFP and RFP fluorescence images were sequentially captured with a CCD camera (Hamamatsu Photonics, Shizuoka, Japan). Image acquisition was controlled by Metamorph software (Molecular Devise). For colchicine treatment, testes from young adult flies were dissected under 50 µmg/ml colchicine in BRB50 buffer, and spread under mineral oil. For cytochalasin D treatment, testes from adult flies were dissected under 10 µg/ml cytochalasin D in BRB50 buffer, and spread under mineral oil. We used DMSO (Sigma) as a control. Testes squashes to evaluate onion-stage spermatids were performed according to the protocol of [33], and viewed under phase-contrast microscopy.

### Immunofluorescence

Testis cells were fixed according to the method of Inoue et al. (2004). The following primary antibodies were used: anti-Orbit [33], anti-Zip (a gift from K. Prehoda), anti-Pav (D. Glover), anti-anillin (C. Field), anti-Pebble (A. Müller, [66]), and anti-RacGAP50C (M. Murray). MTs were visualized by immunostaining with anti-β-tubulin (GTU-88, Sigma-Aldrich, St. Louis, MO), expression of GFP-β-tubulin [33], or RFP-β-tubulin (in this study). F-Actin was visualized with Alexa-594 phalloidin, or expression of GFP-actin5C, RFP-actin5C, or CFP-β-actin. All secondary antibodies were commercially obtained. Images were processed and merged in pseudocolor using MetaMorph version 7.6 (Molecular Devises).

## Supporting Information

Table S1 Quantification of meiotic defects appeared in onion stage spermatids from males expressing dsRNA of genes related to cytokinesis.
(DOC)

Table S2 A requirement of cytoskeletal proteins, contractile ring proteins and their regulatory proteins for each step of myosin II localization in cytokinesis of meiotic divisions in *Drosophila* male.
(DOCX)

File S1 **Supporting figures.** **Figure S1.** Time-lapse observation of myosin II in spermatocytes with expression of GFP-Sqh in the presence of colchicine. Left column; tubulin in green, myosin in red. (A) MLC is recruited along the cell cortex (large arrow, anaphase onset; t = 0 min). The recruited MLC becomes to be accumulated at the equatorial cortex soon after peripheral microtubules become attached (small arrow, t = 10 min). Note that the myosin foci are not yet evident at this time. (B) Time-lapse observation of myosin II in the presence of colchicine (colchicine addition; t = 0 min). Both astral and spindle MTs begin to degrade and furrow ingression has not started. The initial cortical recruitment of myosin II can be observed (arrows), but their accumulation toward the equatorial cortex is inhibited (open arrowheads). Scale bars  = 10 µm. **Figure S2.** Dynamic cellular localization of two centralspindlin components, Pebble and Rho1 during male meiosis. (A–D) Time-lapse observation of GFP-tagged protein (green) and RFP-tubulin (red) during male meiosis I. Anaphase onset was set at t = 0 min. (A, B) Time-lapse observation of Pavarotti (A, green) or GFP-RacGAP50C (B, green) in spermatocytes at early anaphase (t = 0 min). RacGAP50C appears to be accumulated at the spindle mid-zone and the contractile ring. (C) Time-lapse observation of GFP-Pebble (green) during male meiosis I. Note that faint Pebble foci appeared at the equatorial cortex (arrowheads, t = 10 min). Pebble proteins seemed to accumulate along CS MTs and on the ring structure corresponding to the contractile ring. (D) Time-lapse observation of GFP-Rho1 (green) during male meiosis I. Note that any Rho1 foci as shown in Fig. S2A failed to be found at the CF, although weak distribution appeared along the cell cortex. Scale bars  = 10 µm. **Figure S3.** Depletion of pebble affects the formation of peripheral and interior CS MTs and CF ingression. (A, B) Microtubule dynamics by using expression of RFP-tubulin as a probe (anaphase onset; t = 0 min). The right column indicates a series of phase contrast images to monitor CF ingression. (A) Normal control. (B) Simultaneous expression of dsRNA for pebble failed to demonstrate significant defects in peripheral microtubules, but the interior CS is ultimately disrupted (t = 30 min). (C) Testis-specific induction of dsRNA for pebble results in a considerable reduction (0.13 of the Pebble protein in wild-type extracts) of the protein in the testis extracts. (D) Testis-specific induction of dsRNA for pav results in a half reduction (0.55 of the Pav protein in wild-type extracts) of the protein in the testis extracts. **Figure S4.** Time-lapse observation of MLC-GFP in a living primary spermatocyte from *orbit^7^* mutant male. Time in each panel is shown in minutes relative to the onset of anaphase I (t = 0 min) and simultaneously acquired phase contrast micrographs are presented in the right column. This spermatocyte displays a distinct cortical accumulation of Orbit at the equatorial region (arrowheads, t = 10 min), although the Orbit band constructed at the cell mid-zone is disrupted and disappears (t = 30 min). This type of abnormal myosin dynamic was observed in 5 out of 8 mutant spermatocytes examined.
(PDF)

Movie S1 **A movie for the time-lapse experiment presented in** **Fig. 1A** **.** We simultaneously obtained time-lapse images of GFP-Tubulin fluorescence and mRFP-Actin fluorescence for spermatocytes undergoing meiosis I. These images are animated in a combined movie (GFP-Tubulin in green, mRFP-Actin in red).
(WMV)

Movie S2 **The mRFP-Actin images are in a separated movie, Movie S2.**
(WMV)

Movie S3 **A movie for the time-lapse experiment presented in** **Fig. 1C** **.** Time-lapse images are animated in a combined movie (GFP-Tubulin in green, mRFP-Sqh in red).
(WMV)

Movie S4 **A movie for the time-lapse experiment presented in** **Fig. 2A** **.** Time-lapse images of a Pav-depleted cell are animated in a combined movie (GFP-tubulin in green, mRFP-MLC in red).
(WMV)

Movie S5 **A movie for the time-lapse experiment presented in** **Fig. 2B** **.** Time-lapse images of a Pbl-depleted cell are animated in a combined movie (mRFP-tubulin in green, GFP-Sqh in red).
(WMV)

Movie S6 **A movie for the time-lapse experiment presented in** **Fig. 3B** **.** Time-lapse images of a control cell are animated in a combined movie (mRFP-Orbit in green, GFP-Sqh in red).
(WMV)

Movie S7 **A movie for the time-lapse experiment presented in** **Fig. 3C** **.** Time-lapse images of an *orbit^7^* mutant cell are animated in a combined movie (mRFP-Tubulin in green, GFP-Sqh in red).
(WMV)

Movie S8 **A movie for the time-lapse experiment presented in** **Fig. 6B** **.** We obtained time-lapse images of GFP-Orbit fluorescence for a MHC-depleted spermatocyte undergoing meiosis I.
(WMV)

Movie S9 **A movie for the time-lapse experiment presented in** **Fig. 6C** **.** We obtained time-lapse images of GFP-Orbit fluorescence for an anillin-depleted spermatocyte undergoing meiosis I.
(WMV)

Movie S10 **A movie for the time-lapse experiment presented in** **Fig. 7C** **.** We obtained time-lapse images of GFP-Orbit fluorescence for a Feo-depleted spermatocyte undergoing meiosis I. Time-lapse images of the bottom cell were used as still images in Fig. 7C.
(WMV)

Movie S11 **A movie for the time-lapse experiment presented in** **Fig. 7F** **.** We obtained time-lapse images of GFP-Orbit fluorescence for a Pebble-depleted spermatocyte undergoing meiosis I.
(WMV)
