# Supplementary material for: Orbit/CLASP Is Required for Myosin Accumulation at the Cleavage Furrow in Drosophila Male Meiosis
Source: PLoS One. 2014 May 21;9(5):e93669. doi: 10.1371/journal.pone.0093669 (PMC4029619; doi:10.1371/journal.pone.0093669)
Supplement: Table S1 — Quantification of meiotic defects appeared in onion stage spermatids from males expressing dsRNA of genes related to cytokinesis. (DOC) [file pone.0093669.s001.doc]

| **Table S1.** Quantification of meiotic defects appeared in onion stage spermatids from males expressing dsRNA of genes related to cytokinesis | | | | | | | | | | | | |
| --- | --- | --- | --- | --- | --- | --- | --- | --- | --- | --- | --- | --- |
| Category | Symbol | Stock Number | N*^1^ | Nebenkern-to-nuclei ratio (cells) | | | | | | | | macro /micronuclei  ( cells)*^3^ |
|  |  |  |  | Normal | Abnormal*^2^ | | | | | | |  |
|  |  |  |  |  | 1/0 | 1:1 | 1:2 | 1:3 | 1:4 | 1:>5 | N |  |
| control | yw |  |  |  |  |  |  |  |  |  |  |  |
| A | **dia** | v103914 | 609 | 0 | 0 | 0 | 0 | 0 | 609 | 0 | 609 | 0 |
|  |  | v20518 | 694 | 81 | 0 | 0 | 89 | 21 | 503 | 0 | 613 | 0 |
| A | **act5C** | v1351 | 551 | 11 | 3 | 0 | 63 | 30 | 422 | 22 | 540 | 5 |
|  |  | v101438 | 354 | 41 | 0 | 0 | 56 | 0 | 257 | 0 | 313 | 0 |
| B | **act42A** | v1350 | 647 | 0 | 0 | 0 | 20 | 0 | 425 | 202 | 647 | 202 |
| A | **anillin** | V33465 | 472 | 8 | 0 | 0 | 87 | 15 | 362 | 0 | 464 | 0 |
|  |  | v104674 | 579 | 17 | 0 | 0 | 80 | 10 | 472 | 0 | 562 | 0 |
| A | **zip** | V7819 | 980 | 0 | 0 | 0 | 70 | 23 | 887 | 0 | 980 | 0 |
| B  A | **sqh** | V7916 | 456 | 0 | 0 | 0 | 1 | 9 | 162 | 284 | 456 | 284 |
|  |  | v109493 | 942 | 0 | 0 | 0 | 15 | 4 | 923 | 0 | 942 | 0 |
| A | **pebble** | v12384 | 333 | 0 | 0 | 0 | 0 | 0 | 333 | 0 | 333 | 0 |
|  |  | v102244 | 692 | 0 | 0 | 0 | 0 | 0 | 692 | 0 | 692 | 0 |
| A | **Pav-KLP** | v46137 | 543 | 0 | 0 | 0 | 0 | 0 | 543 | 0 | 543 | 0 |
|  |  | v110330 | 717 | 0 | 0 | 0 | 0 | 0 | 717 | 0 | 717 | 0 |
| A | **rhoA** | BL9909 | 453 | 53 | 0 | 0 | 3 | 0 | 397 | 0 | 400 | 0 |
|  |  | BL29002 | 328 | 0 | 0 | 0 | 0 | 0 | 328 | 0 | 328 | 0 |
|  |  | v12734 | 558 | 280 | 0 | 0 | 11 | 0 | 267 | 0 | 278 | 0 |
| D |  | v109420 | *4 |  |  |  |  |  |  |  |  |  |
| A | **mtl** | v108427 | 811 | 228 | 0 | 0 | 59 | 21 | 503 | 0 | 583 | 0 |
| A | **KLP3A** | v35975 | 791 | 203 | 0 | 0 | 183 | 0 | 405 | 0 | 588 | 0 |
|  |  | v104682 | 1158 | 1021 | 0 | 0 | 92 | 0 | 45 | 0 | 137 | 0 |
| A | **KLP38B** | v108138 | 593 | 97 | 0 | 0 | 178 | 0 | 303 | 15 | 496 | 15 |
| A | **feo** | v7834 | 399 | 5 | 0 | 0 | 106 | 0 | 288 | 0 | 394 | 0 |
|  |  | NIG11207R-2 | 708 | 295 | 0 | 0 | 75 | 0 | 338 | 0 | 413 | 0 |
| A | **tsr** | v110599 | 381 | 0 | 0 | 0 | 54 | 0 | 322 | 5 | 381 | 5 |
| A | **polo** | BL33042 | 745 | 161 | 0 | 0 | 251 | 0 | 333 | 0 | 584 | 0 |
| D |  | v20177 | *6 |  |  |  |  |  |  |  |  |  |
| A | **Incenp** | NIG12165R-3 | 550 | 0 | 0 | 0 | 19 | 17 | 503 | 11 | 550 | 0 |
| B |  | v17044 | 250 | 0 | 3 | 26 | 42 | 10 | 78 | 91 | 250 | 130 |
| D |  | v101123 | *6 |  |  |  |  |  |  |  |  |  |
| A | **AuroraB** | NIG6620R-2 | 1132 | 1027 | 0 | 0 | 0 | 0 | 105 | 0 | 105 | 0 |
| B |  | v35107 | 291 | 0 | 0 | 0 | 107 | 74 | 101 | 9 | 291 | 70 |
| D |  | v104051 | *6 |  |  |  |  |  |  |  |  |  |
| B | **CLIP190** | v107176 | 446 | 132 | 0 | 23 | 83 | 30 | 141 | 37 | 314 | 104 |
| B | **KLP67A** | v108852 | 554 | 0 | 0 | 6 | 14 | 107 | 199 | 228 | 554 | 554 |
| C*^6^ | **Gl** | v3785 | 787 | 601 | 73 | 109 | 4 | 0 | 0 | 0 | 186 | 113 |
| C | **shot wing** | v101559 | 553 | 278 | 121 | 131 | 23 | 0 | 0 | 0 | 275 | 154 |
|  |  | v48333 | 710 | 384 | 143 | 139 | 44 | 0 | 0 | 0 | 326 | 183 |
| C | **cut up** | v109084 | 981 | 745 | 111 | 109 | 16 | 0 | 0 | 0 | 236 | 125 |
| C | **KLP61F** | v52548 | 1722 | 423 | 111 | 200 | 396 | 293 | 176 | 123 | 1299 | 1188 |
|  |  | v109280 | 1429 | 484 | 92 | 210 | 297 | 177 | 66 | 103 | 945 | 853 |
| C | **cenp meta** | v35081 | 419 | 130 | 85 | 162 | 38 | 4 | 0 | 0 | 289 | 204 |
| C | **Dhc64C** | BL28749 | 453 | 341 | 0 | 16 | 13 | 42 | 10 | 31 | 112 | 112 |

All genotypes are w−. N is number of onion-stage spermatids scored. Testes from 10 males were scored per genotype. Type A: cytokinesis defects without or with less frequent chromosome segregation defects appeared in spermatids with expression of dsRNA. Type B: both cytokinesis and chromosome segregation defects appeared in spermatids with expression of dsRNA. *^1^N:Total spermatids scored. *^2^ Spermatids at onion stage were examined about Nebenkern-to-nuclei ratio. Spermatids with abnormally sized Nebenkerns or their abnormal morphology were also felt into this category. Cells appeared in second column of the abnormal category showed normal Nebenkern-to-nuclei ratio (1:1) but they carried abnormally shaped Nebenkerns. *^3^All abnormal spermatids carrying larger or smaller nuclei were separately counted and summarized in this category. *^4^No spermatids at onion stage. *^5^Type C: chromosome segregation defects without a failure of cytokinesis appeared in spermatids with ds RNA expression. *^6^Less numbers of spermatids were observed within single cysts with expression of dsRNA for type D genes.

Abbreviations before stock numbers indicate stocks from the following stock centers: ‘v ‘ from VDRC, ‘BL’ from Bloomington Drosophila Stock Center, ‘NIG’ from the National Institute of Genetics.
