# Supplementary material for: Orbit/CLASP Is Required for Myosin Accumulation at the Cleavage Furrow in Drosophila Male Meiosis
Source: PLoS One. 2014 May 21;9(5):e93669. doi: 10.1371/journal.pone.0093669 (PMC4029619; doi:10.1371/journal.pone.0093669)
Supplement: Table S2 — A requirement of cytoskeletal proteins, contractile ring proteins and their regulatory proteins for each step of myosin II localization in cytokinesis of meiotic divisions in Drosophila male. (DOCX) [file pone.0093669.s002.docx]

**Table S2.** A requirement of cytoskeletal proteins, contractile ring proteins and their regulatory proteins for each step of myosin II localization in cytokinesis of meiotic divisions in *Drosophila* male.

| Myosin localization  Requirement | Cell cortex loading | Accumulation at the presumptive CF site | CR |
| --- | --- | --- | --- |
| Myosin II | + | + | + |
| F-actin | -* | -* | +* |
| Microtubules | - | + | + |
| Anillin | - | - | + |
| Rho1 | - | + | + |
| Pbl | - | - | + |
| Pav | - | - | + |
| Orbit | - | + | + |

+, essential, -:dispensable, ND:not determined

*A requirement of F-actin for myosin II localization was not examined in male meiosis but in mitosis using S2 cultured cells.
