# Supplementary figures and images for: Orbit/CLASP Is Required for Myosin Accumulation at the Cleavage Furrow in Drosophila Male Meiosis
Source: PLoS One. 2014 May 21;9(5):e93669. doi: 10.1371/journal.pone.0093669 (PMC4029619; doi:10.1371/journal.pone.0093669)

Supplementary Fig S1

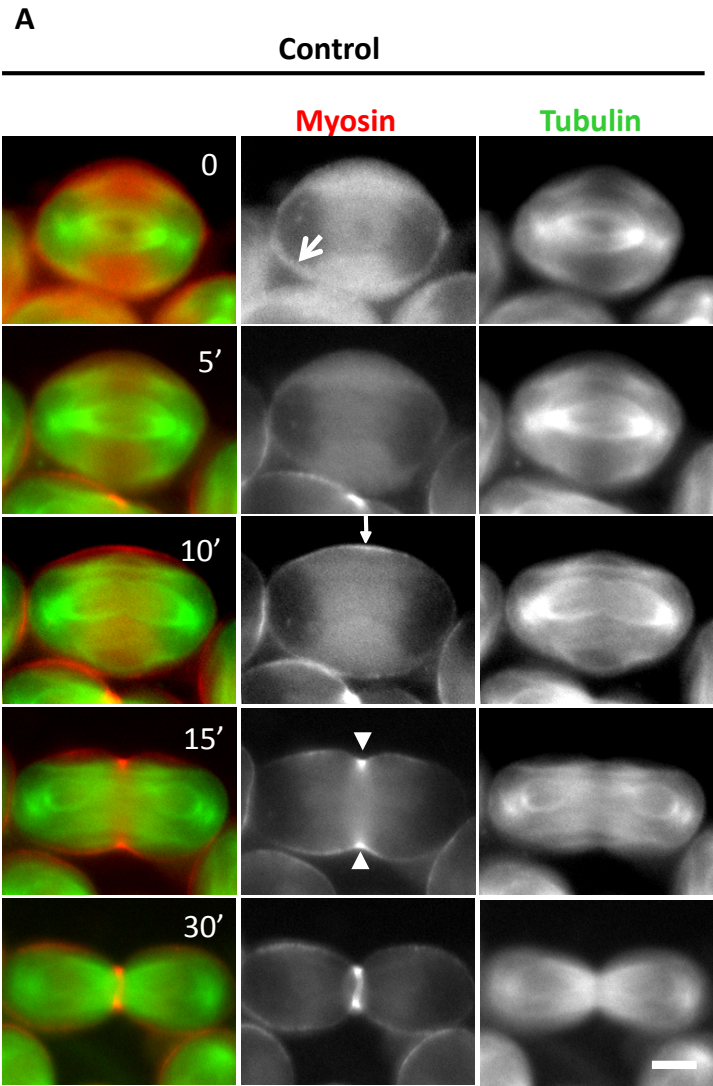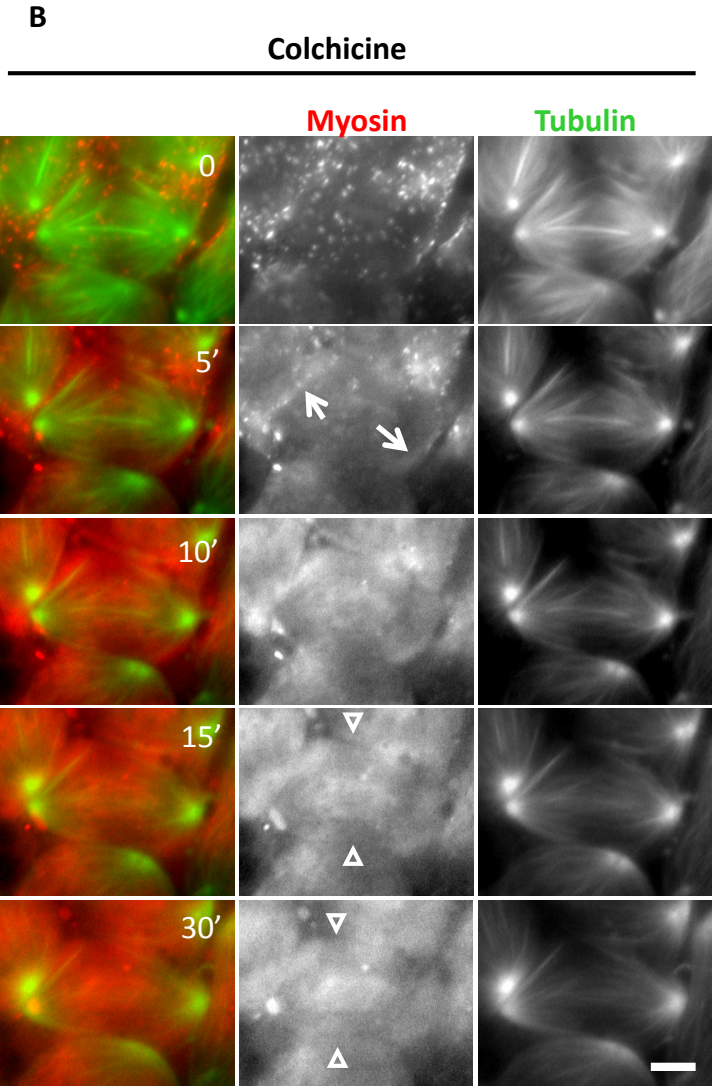

Supplementary Fig S2

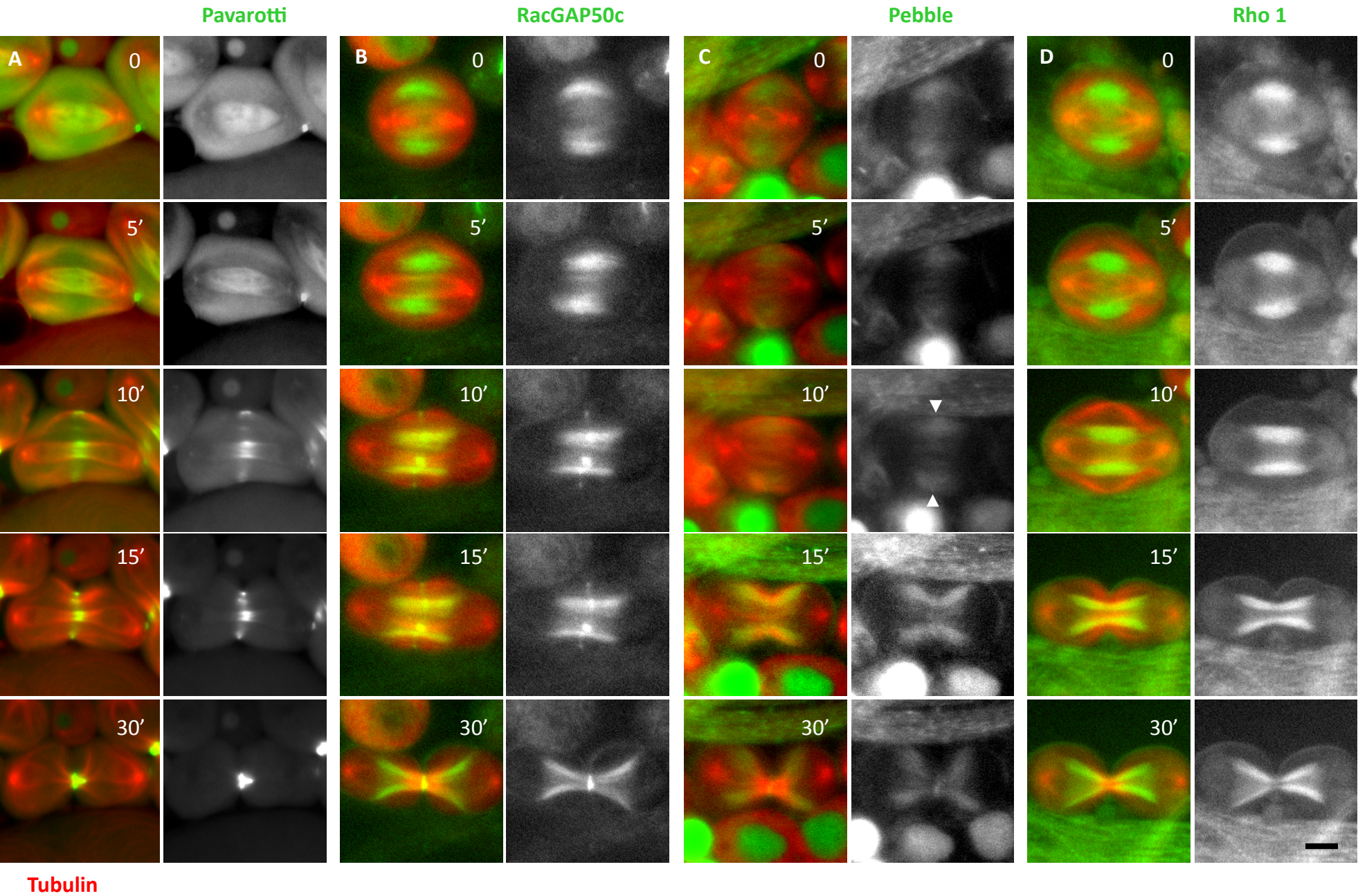

Supplementary Fig S3

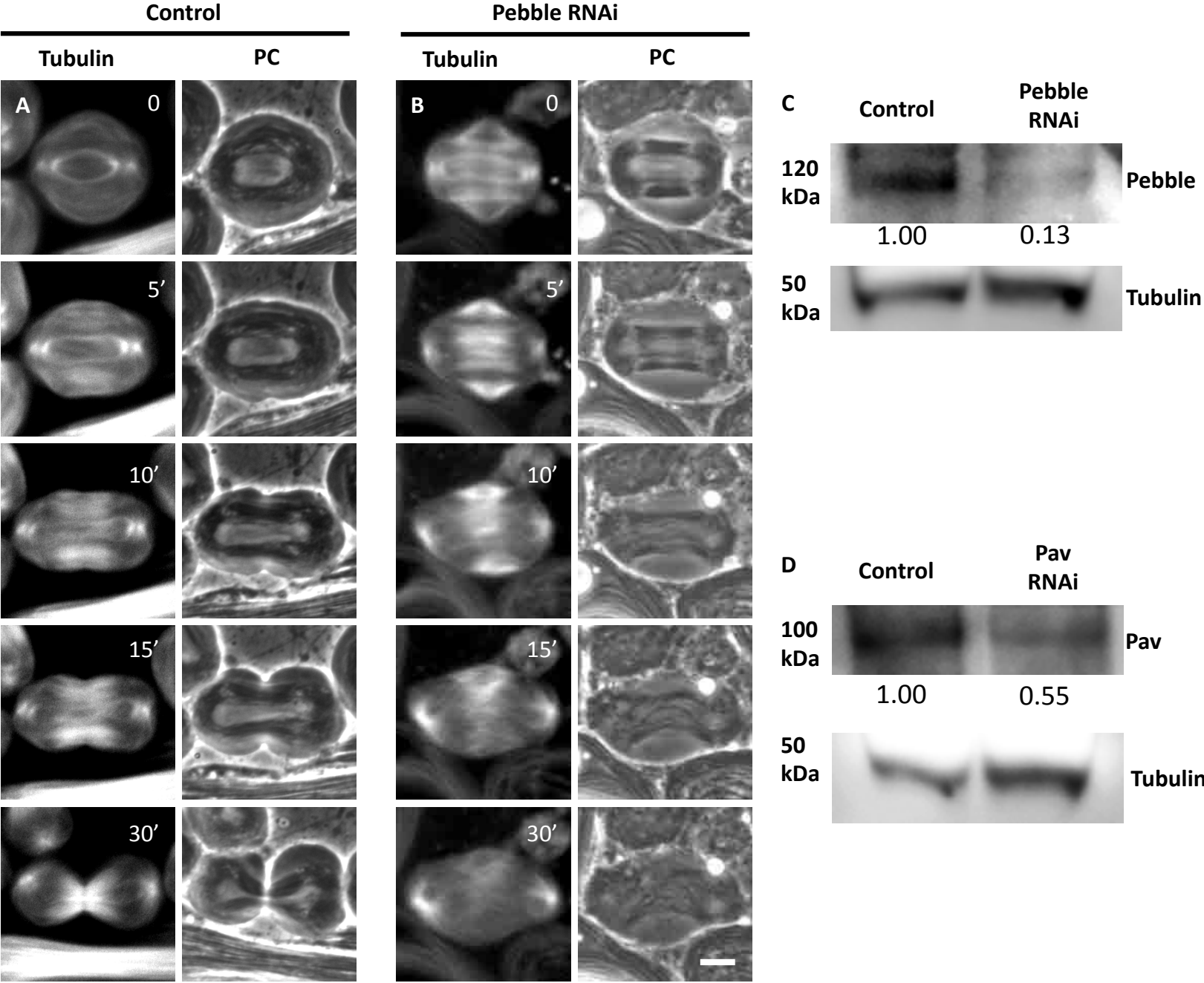

Supplementary Fig S4

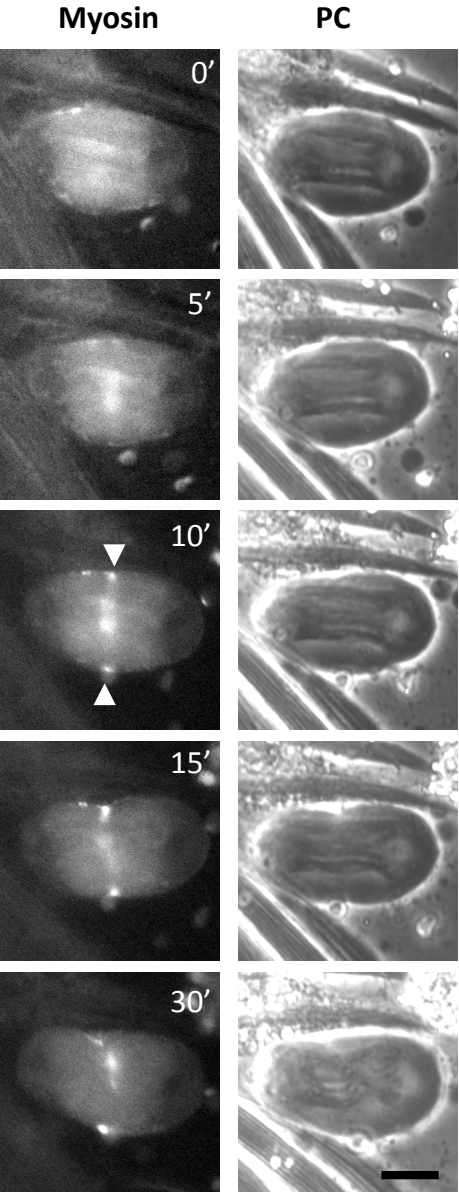

Supplement: File S1 — Supporting figures. Figure S1. Time-lapse observation of myosin II in spermatocytes with expression of GFP-Sqh in the presence of colchicine. Left column; tubulin in green, myosin in red. (A) MLC is recruited along the cell cortex (large arrow, anaphase onset; t = 0 min). The recruited MLC becomes to be accumulated at the equatorial cortex soon after peripheral microtubules become attached (small arrow, t = 10 min). Note that the myosin foci are not yet evident at this time. (B) Time-lapse observation of myosin II in the presence of colchicine (colchicine addition; t = 0 min). Both astral and spindle MTs begin to degrade and furrow ingression has not started. The initial cortical recruitment of myosin II can be observed (arrows), but their accumulation toward the equatorial cortex is inhibited (open arrowheads). Scale bars = 10 µm. Figure S2. Dynamic cellular localization of two centralspindlin components, Pebble and Rho1 during male meiosis. (A–D) Time-lapse observation of GFP-tagged protein (green) and RFP-tubulin (red) during male meiosis I. Anaphase onset was set at t = 0 min. (A, B) Time-lapse observation of Pavarotti (A, green) or GFP-RacGAP50C (B, green) in spermatocytes at early anaphase (t = 0 min). RacGAP50C appears to be accumulated at the spindle mid-zone and the contractile ring. (C) Time-lapse observation of GFP-Pebble (green) during male meiosis I. Note that faint Pebble foci appeared at the equatorial cortex (arrowheads, t = 10 min). Pebble proteins seemed to accumulate along CS MTs and on the ring structure corresponding to the contractile ring. (D) Time-lapse observation of GFP-Rho1 (green) during male meiosis I. Note that any Rho1 foci as shown in Fig. S2A failed to be found at the CF, although weak distribution appeared along the cell cortex. Scale bars = 10 µm. Figure S3. Depletion of pebble affects the formation of peripheral and interior CS MTs and CF ingression. (A, B) Microtubule dynamics by using expression of RFP-tubulin as a probe (anapha [file pone.0093669.s003.pdf]
